# Genome Wide Association Study Identifies 20 Novel Promising Genes Associated with Milk Fatty Acid Traits in Chinese Holstein

**DOI:** 10.1371/journal.pone.0096186

**Published:** 2014-05-23

**Authors:** Cong Li, Dongxiao Sun, Shengli Zhang, Sheng Wang, Xiaoping Wu, Qin Zhang, Lin Liu, Yanhua Li, Lv Qiao

**Affiliations:** 1 Key Laboratory of Animal Genetics and Breeding of Ministry of Agriculture, National Engineering Laboratory of Animal Breeding, College of Animal Science and Technology, China Agricultural University, Beijing, China; 2 Beijing Dairy Cattle Center, Beijing, China; The University of Chicago, United States of America

## Abstract

Detecting genes associated with milk fat composition could provide valuable insights into the complex genetic networks of genes underling variation in fatty acids synthesis and point towards opportunities for changing milk fat composition via selective breeding. In this study, we conducted a genome-wide association study (GWAS) for 22 milk fatty acids in 784 Chinese Holstein cows with the PLINK software. Genotypes were obtained with the Illumina BovineSNP50 Bead chip and a total of 40,604 informative, high-quality single nucleotide polymorphisms (SNPs) were used. Totally, 83 genome-wide significant SNPs and 314 suggestive significant SNPs associated with 18 milk fatty acid traits were detected. Chromosome regions that affect milk fatty acid traits were mainly observed on BTA1, 2, 5, 6, 7, 9, 13, 14, 18, 19, 20, 21, 23, 26 and 27. Of these, 146 SNPs were associated with more than one milk fatty acid trait; most of studied fatty acid traits were significant associated with multiple SNPs, especially C18:0 (105 SNPs), C18 index (93 SNPs), and C14 index (84 SNPs); Several SNPs are close to or within the *DGAT1*, *SCD1* and *FASN* genes which are well-known to affect milk composition traits of dairy cattle. Combined with the previously reported QTL regions and the biological functions of the genes, 20 novel promising candidates for C10:0, C12:0, C14:0, C14:1, C14 index, C18:0, C18:1n9c, C18 index, SFA, UFA and SFA/UFA were found, which composed of *HTR1B*, *CPM*, *PRKG1*, *MINPP1*, *LIPJ*, *LIPK*, *EHHADH*, *MOGAT1*, *ECHS1*, *STAT1*, *SORBS1*, *NFKB2*, *AGPAT3*, *CHUK*, *OSBPL8*, *PRLR*, *IGF1R*, *ACSL3*, *GHR* and *OXCT1*. Our findings provide a groundwork for unraveling the key genes and causal mutations affecting milk fatty acid traits in dairy cattle.

## Introduction

Fat is the major energy substance in milk and more than 50% milk total energy comes from milk fat, which accounts 3–5% of milk contents. Fat nutrition value depends on fatty acids. Monounsaturated fatty acids (MUFA) have a favourable effect on human health because of its cholesterol-declining properties. Polyunsaturated fatty acids (PUFA) of the n-6 and n-3 series are essential nutrients that exert an important influence on plasma lipids and serve cardiac and endothelial functions for prevention and treatment of coronary heart diseases [1]. Conjugated linoleic acid (CLA) has effects on bone formation and the immune system as well as fatty acids and lipid metabolism and gene expression in numerous tissues [2]. Saturated fatty acids (SFA) lead to increase the concentration of low density lipoprotein (LDL) cholesterol and cause cardio cerebral vascular disease [3]. Therefore, changing the proportions of dietary fat by decreasing SFA and increasing MUFA and PUFA is vital to Human health. It is suggested that the ideal balance would seem to approximate 1∶1.3∶1 for SFA∶MUFA∶PUFA [4].

From the genetics point of view, milk fatty acids are complex traits influenced by non-genetic factors, such as breed, herd, stage of lactation, etc [5], [6] and genetic factors [7]. Bovine milk fatty acids have been found to be heritable, with heritability estimates ranging from 0.22 to 0.71 [8], [9]. Short and medium chain C4 to C16 saturated and monounsaturated fatty acids, which are synthesized de novo in the mammary gland, have moderate to high heritability (0.4–0.6) [8], [9]. Long chain fatty acids (above C16) are derived from circulating plasma lipids, whereas have low to moderate heritability (about 0.2) [8], [9]. Identifying genes and loci responsible for the genetic variation is expected to contribute greatly to our understanding of milk fatty acids synthesis, and to develop a marker-assisted selection to improve fatty acids in dairy breeding program in future. In the past few years, candidate gene and quantitative trait locus (QTL) mapping approaches have been implemented to detect genes or QTLs for milk fatty acid traits. A few promising loci, e.g. *DGAT1* p.Lys232Ala and *SCD1* p.Ala293Val [10], [11], [12] and a large number of significant or suggestive genomic regions [13], [14] were identified. Although the above two methods have got a few prominent findings, identification of causal mutations is still a challenge due to the commonly existing limitations [15].

At present, genome-wide association study (GWAS) has become a powerful strategy to identify genetic variants associated with complex traits. Since the first GWAS was published in 2005 [16], a great number of relative studies were conducted in human and domestic animals. Of them, several GWASs have been applied to detect genes or loci for milk production traits [17], [18], [19], conformation traits [19], reproduction traits [20], [21], healthy traits [22], [23], *etc*, in dairy and beef cattle. However, only studies have been carried out for fatty acids in Dutch dairy cattle [24], [25]. We herein performed a GWAS for 22 milk fatty acid traits in a Chinese Holstein population to identify genes and chromosome segments with large effects on such traits.

## Materials and Methods

The milk samples were collected along the regular quarantine inspection of the farms. The whole procedure for sample collection was carried out in strict accordance with the protocol approved by the Animal Welfare Committee of China Agricultural University (Permit Number: DK996).

### Phenotypic data and traits

The Chinese Holstein population in this study comprised 784 cows, the daughters of 21 sire families. All cows in this study were from 18 farms of the Beijing Sanyuan Dairy Farm Center, where routine standard performance test, i.e. Dairy Herd Improvement system (DHI) have been carried out since 1999. A total of 50 ml milk sample was collected for each cow from the DHI laboratory of the Beijing Dairy Cattle Center during November to December, 2012. The procedure of milk sample collection was carried out corresponding to DHI sampling (dairy herd improvement). After DHI measure, the remaining milk samples were taken back to the laboratory within 4°C cooler and then stored at −20°C.

Phenotypic values of 16 kinds of main milk fatty acids were measured by gas chromatography at the Ministry of Agriculture Feed Industry Centre of China (http://www.mafic.ac.cn/intro/default.asp), which included SFA of C8:0, C10:0, C12:0, C14:0, C16:0, C18:0, C20:0, C22:0; MUFA of C14:1, C16:1, C18:1n9c; PUFA of CLA (*cis*-9, *trans*-11 C18:2), C18:3n3, C18:3n6, C18:2n6c and C20:5n3. Before measuring, milk samples should be done with pretreatment. First, total milk fat were extracted from approximately 2 ml of each milk sample. The specific procedure was as follows: 2 ml milk was mixed with 4 ml solution of N-hexane/isopropyl alcohol (3∶2) and 2 ml solution of Na_2_SO_4_, and centrifuged at 3,000×*g* for 20 min. The upper layer was collected into 20 ml hydrolysis tube and 200 µl of C19:0 methyl ester as the internal standard was mixed, and then the extracted fat was dried under nitrogen. Methyl esters of fat were performed in the next step. 2 ml of NaOCH3/Methanol was put into the above hydrolysis tube for 15 min water bath under 50°C, and was mixed with 2 ml of hydrochloric acid/methanol solution (1∶10) for 1.5 h water bath under 80°C. After the temperature fell to room temperature level, 3 ml of water and 6 ml of n-hexane were put into above hydrolysis tube, mixed, vortexed, and stratified. The upper layer was collected and dried under nitrogen, and finally dissolved in 1 ml of n-hexane. 1 ml methyl esters of fatty acids were prepared to be determined by gas chromatography using a gas chromatograph (6890N, Agilent) equipped with a flame-ionization detector and a high polar fused silica capillary column (SP™-2560, 100 m×0.25 mm ID, 0.20 µm film). About 1 µl sample was injected under the following gas chromatography conditions: Helium was used as the carrier gas at a flow of 45 ml/min. The split ratio was 100∶1. The oven temperature was programmed at 100°C and held for 10 min, then increased to 160°C at a rate of 6°C/min, held for 10 min, increased to 200°C with 5°C/min, held for 20 min, increased to 240°C at a rate of 4°C/min and held for 12 min. Both the injector temperature and the detector temperature were set on 260°C. Individual fatty acids were identified and quantified by comparing the methyl ester chromatograms of the milk fat samples with the chromatograms of pure fatty acids methyl ester standards (Supelco™ 37 Component FAME Mix), and were measured as the weight proportion of total fat weight (wt/wt%).

Based on the phenotypes of 16 milk fatty acids, 6 additional traits were obtained including SFA, UFA, SFA/UFA (the ratio of SFA to UFA), C14 index, C16 index and C18 index. The 3 indices were calculated as 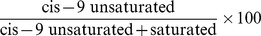 [26].

The descriptive statistics of these 22 fatty acid traits are presented in Table 1. Both SFA and UFA accounted for approximately 96% (wt/wt) of total fat.

**Table 1:** Descriptive statistics of the 22 fatty acid traits in Chinese Holstein.

Traits	No. cows	Mean	Standard deviation	Variable coefficient^a^	Maximum	Minimum
C8:0	784	0.578	0.223	38.501	1.200	0.128
C10:0	784	2.193	0.428	19.538	3.286	0.967
C12:0	784	2.865	0.543	18.943	4.513	1.194
C14:0	784	9.892	1.285	12.988	13.546	5.808
C14:1	784	0.835	0.221	26.429	1.598	0.339
C16:0	784	32.665	1.998	6.116	39.995	25.182
C16:1	784	1.656	0.376	22.710	3.735	0.158
C18:0	784	12.169	1.761	14.472	17.367	7.401
C18:1n9c	784	28.571	2.814	9.849	38.289	18.873
C18:2n6c	784	4.002	0.462	11.548	5.895	2.264
C18:3n6	784	0.098	0.064	65.520	0.457	0.003
C18:3n3	784	0.417	0.065	15.681	0.627	0.008
CLA	784	0.404	0.094	23.276	0.797	0.050
C20:0	784	0.163	0.047	28.981	0.376	0.006
C20:5n3	784	0.041	0.021	51.366	0.180	0.012
C22:0	784	0.054	0.027	49.351	0.289	0.003
C14 index	784	7.763	1.677	21.596	14.624	3.460
C16 index	784	4.831	1.071	22.179	9.118	0.420
C18 index	784	70.144	3.335	4.754	80.298	55.891
SFA	784	62.134	3.066	4.934	72.604	47.670
UFA	784	36.481	3.044	8.345	46.678	26.156
SFA/UFA	784	1.722	0.225	13.092	2.776	1.113

Note:

a Variable coefficient calculated as the ratio of standard deviation (SD) to the mean multiplied by 100.

### Genotypes and quality control

The cows were genotyped using the Illumina BovineSNP50 BeadChip (Illumina Inc., San Diego, CA, US), of which, some individuals were genotyped with the 54K chip version1 containing 54,001 SNPs, and others were genotyped with the 54K chip version 2 including 54,609 SNPs. After genotype imputation by BEAGLE software (http://faculty.washington.edu/browning/beagle/beagle.html), the common SNP markers in both version chips were used in this study, as a result, the total number of SNPs in the panel was 52,340. The SNP positions were based on the bovinegenome assembly UMD_3.1.66 (http://www.ncbi.nlm.nih.gov/genome/guide/cow/).

The quality control procedure was as follows, 20 daughters were excluded due to low call rate (<90%), leading to 764 daughters remaining for the association analysis. On the other hand, 11,736 SNPs were removed for falling to meet the following requirements: 652 SNPs with <90% genotype call rate, 10,798 SNPs with a minor allele frequency (MAF) <0.05, 286 SNPs with extreme value of Hardy-Weinberg equilibrium statistics (P<10^−6^). Eventually, 40,604 SNPs passed these quality control filters, which was 77.6% of the SNPs in the panel. The average distance between adjacent markers was quite constant among different chromosomes. The shortest average distance was 56 kb on BTA25, and the longest average distance was 75 kb on BTA5 (except for 198 kb on BTAX).

### Statistical analysis

The statistical tests followed a two-step analysis. For the first step, phenotypic values were corrected for fixed non-genetic effects by using SAS 9.1 general linear model (GLM) procedure. The statistical model was: 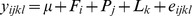, where *y*_ijkl_ was the unadjusted phenotype; *μ* was the overall mean; *F_i_* was the fixed effect of farm; *P_j_* was the fixed effect of parity; *L_k_* was the fixed effect of stage of lactation; *e*_ijkl_ was the random residual. In the second step, genome-wide association analyses were performed with quantitative trait procedure (additive model)of the PLINK software (v1.07) [27], and empirical p-values estimated based on the Wald-statistic. Individual pedigree of three generations was applied.

Manhattan plots of genome-wide association analyses were produced with R2.15.1 software (http://www.r-project.org/).

### Significance level

Bonferroni correction was applied to adjust for multiple testing from the number of SNPs detected. A significant SNP at the genome-wise significance level was declared if a raw *P* value (unadjusted)<0.05/*N*, *N* is the number of SNP markers tested in analyses [28]. In the present study, Bonferroni genome-wise significance was 1.23E-06 (0.05/40604). As the Bonferroni correction threshold levels were strict and may lead to high false negatives, we calculated suggestive significant association threshold *P*-value as previously described [29], which was 2.46E-05 (1/40604).

## Results

The global view of *P*-values for all SNPs of each trait was shown in Additional file 1. In total, 83 genome-wise significant SNPs (*P*<1.23E-06) and 314 suggestive significant SNPs (*P*<2.46E-05) were detected for 22 milk fatty acids on all chromosomes, ranged from 3 on BTA3, 4, 22 to 119 on BTA26 (Tables 2–6). For most of the studied fatty acids, significant associations were detected with more than one SNP, especially C14:1 (67 SNPs: 34 genome-wide, 33 suggestive), C18:0 (105 SNPs: 13 genome-wide, 92 suggestive), C14 index (84 SNPs: 49 genome-wide, 35 suggestive) and C18 index (93 SNPs: 14 genome-wide, 79 suggestive) (Table 7). The most significant SNP (BTB-00931481) was associated with both C14 index (*P* = 6.91E-17) and C14:1 (*P* = 7.08E-13) on BTA26. The top one common significant SNP (ARS-BFGL-NGS-4783) was associated with SFA, SFA/UFA and UFA. Besides, 146 SNPs were associated with multiple traits, especially ARS-BFGL-NGS-4939 on BTA14 for 9 traits. Further details on these associations are described as follows.

**Table 2:** Genome-wise and suggestive significant SNPs for short- and medium-chain saturated fatty acid traits (SCFA and MCFA).

Trait	Rank^a^	SNP name	Chr.	Position(bp)	Nearest Gene/Candidate Gene	Distance(bp)	Raw P_value	P_value Bonferroni
C12:0	17	BTA-120369-no-rs	0	0	*NA*	NA	1.47E-05	0.599
C14:0	19	BTB-00046603	1	103185339	*SI*	Within	1.06E-05	0.432
C14:0	26	BTB-01201574	1	109284636	*SCHIP1*	Within	1.84E-05	0.745
C10:0	4	ARS-BFGL-NGS-23583	1	132049236	*A4GNT*	14386	4.10E-06	0.166
C12:0	9	ARS-BFGL-NGS-23583	1	132049236	*A4GNT*	14386	6.09E-06	0.247
C10:0	15	BTB-00059412	1	132069357	*A4GNT*	1094	1.62E-05	0.658
C10:0	10	ARS-BFGL-NGS-37095	1	132497074	*SOX14*	46530	1.23E-05	0.500
C10:0	17	BTA-51403-no-rs	1	132518716	*SOX14*	68172	1.91E-05	0.774
C10:0	3	ARS-BFGL-NGS-91327	2	12533969	*ZNF804A*	593727	3.70E-06	0.150
C14:0	27	ARS-BFGL-NGS-91327	2	12533969	*ZNF804A*	593727	2.19E-05	0.889
C14:0	17	ARS-BFGL-NGS-117409	2	37256390	*TANC1*	Within	7.04E-06	0.286
C14:0	10	Hapmap42557-BTA-47352	2	38748216	*LOC101907729*	Within	5.01E-06	0.204
C14:0	8	BTB-01053755	2	40313359	*NR4A2*	295672	4.28E-06	0.174
C12:0	21	ARS-BFGL-NGS-30621	2	131539751	*HSPG2*	Within	2.11E-05	0.855
C12:0	16	ARS-BFGL-NGS-20205	3	92427474	*SSBP3*	Within	1.40E-05	0.569
C14:0	20	BTB-01477571	5	43481128	*CNOT2*	Within	1.25E-05	0.509
C14:0	1	Hapmap49848-BTA-106779	5	45089737	***CPM***	Within	**1.58E-07**	**6.42E-03**
C10:0	6	Hapmap49071-BTA-17699	5	92618397	*PIK3C2G*	115016	7.53E-06	0.306
C12:0	10	BTB-01019973	7	79747454	*LOC101904982*	440947	7.57E-06	0.308
C12:0	7	ARS-BFGL-NGS-67383	7	108307729	*EFNA5*	738306	6.00E-06	0.244
C10:0	1	BTB-01556197	9	16892513	***HTR1B***	409905	**5.89E-07**	**2.39E-02**
C12:0	3	BTB-01556197	9	16892513	***HTR1B***	409905	2.66E-06	0.108
C12:0	20	ARS-BFGL-NGS-104719	9	18955509	*HMGN3*	44851	2.10E-05	0.851
C10:0	2	ARS-BFGL-BAC-35400	9	21165167	*FAM46A*	594573	2.28E-06	0.093
C14:0	18	ARS-BFGL-BAC-35400	9	21165167	*FAM46A*	594573	7.23E-06	0.294
C10:0	5	ARS-BFGL-NGS-61979	9	23001645	*UBE3D*	42877	6.06E-06	0.246
C12:0	6	ARS-BFGL-NGS-61979	9	23001645	*UBE3D*	42877	5.66E-06	0.230
C10:0	9	Hapmap39984-BTA-21408	9	28538817	*LOC100848869*	46056	1.10E-05	0.447
C14:0	25	Hapmap41109-BTA-93077	11	42713681	*BCL11A*	358295	1.83E-05	0.743
C14:0	5	ARS-BFGL-BAC-5848	12	68657690	*GPC6*	Within	2.29E-06	0.093
C10:0	20	BTA-37592-no-rs	15	72716471	*LRRC4C*	35911	2.17E-05	0.881
C14:0	23	BTB-00634528	16	31813761	*SMYD3*	Within	1.35E-05	0.549
C14:0	6	UA-IFASA-8132	16	33607353	*C16H1orf100*	7625	2.73E-06	0.111
C10:0	21	BTB-00648332	16	55421856	*PDPN*	Within	2.40E-05	0.973
C14:0	16	ARS-BFGL-NGS-102640	17	5963196	*PET112*	17972	6.63E-06	0.269
C14:0	2	BTB-00669395	17	6266432	*FAM160A1*	Within	2.09E-06	0.085
C14:0	4	Hapmap47945-BTA-41852	17	6295259	*FAM160A1*	Within	2.26E-06	0.092
C14:0	3	BTB-00669586	17	6322271	*FAM160A1*	Within	2.09E-06	0.085
C14:0	13	ARS-BFGL-NGS-20893	17	6669905	*SH3D19*	Within	6.41E-06	0.260
C14:0	14	ARS-BFGL-NGS-115234	18	13136171	*JPH3*	49852	6.48E-06	0.263
C10:0	14	Hapmap23685-BTA-132541	18	54271729	*STRN4/* ***SPHK2***	Within/1442497	1.59E-05	0.647
C10:0	12	UA-IFASA-7471	18	54311149	*SLC1A5/* ***SPHK2***	Within/1403077	1.41E-05	0.571
C10:0	19	ARS-BFGL-NGS-34500	19	15710458	*TMEM132E/* ***ACACA***	81903/1714400	2.11E-05	0.857
C10:0	7	ARS-BFGL-NGS-39328	19	51326750	*CCDC57/* ***FASN***	Within/58172	8.54E-06	0.347
C12:0	2	ARS-BFGL-NGS-39328	19	51326750	*CCDC57/* ***FASN***	Within/58172	**1.16E-06**	**4.71E-02**
C14:0	11	ARS-BFGL-NGS-39328	19	51326750	*CCDC57/* ***FASN***	Within/58172	6.01E-06	0.244
C14:0	21	ARS-BFGL-NGS-87102	20	49859323	*CDH12*	585652	1.26E-05	0.513
C14:0	12	ARS-BFGL-NGS-111676	20	51073910	*CDH12*	Within	6.02E-06	0.244
C12:0	13	Hapmap53927-rs29025287	20	53303717	*CDH18*	106159	1.03E-05	0.417
C12:0	11	BTB-00787949	20	53333822	*CDH18*	76054	8.44E-06	0.343
C14:0	9	BTB-01583562	20	55425112	*LOC784462*	71405	4.71E-06	0.191
C10:0	16	BTA-12468-no-rs	21	9375095	*ARRDC4/* ***IGF1R***	436067/1107002	1.85E-05	0.749
C12:0	12	BTA-12468-no-rs	21	9375095	*ARRDC4/* ***IGF1R***	436067/1107002	1.01E-05	0.411
C10:0	8	BTA-76414-no-rs	21	9528223	*ARRDC4/* ***IGF1R***	589195/1260130	9.90E-06	0.402
C12:0	1	BTA-76414-no-rs	21	9528223	*ARRDC4/* ***IGF1R***	589195/1260130	**3.94E-07**	**1.60E-02**
C10:0	18	ARS-BFGL-NGS-40159	21	21142616	*FANC1/RLBP1/* ***PLIN1***	Within/Within/360208	1.94E-05	0.786
C12:0	14	Hapmap26394-BTA-136497	22	27309084	*CNTN3*	Within	1.05E-05	0.424
C14:0	7	Hapmap26394-BTA-136497	22	27309084	*CNTN3*	Within	3.50E-06	0.142
C10:0	11	Hapmap57060-rs29023510	24	4805759	*FBXO15*	246097	1.33E-05	0.539
C12:0	19	Hapmap57060-rs29023510	24	4805759	*FBXO15*	246097	1.96E-05	0.795
C12:0	4	ARS-BFGL-NGS-78497	24	21330516	*SLC39A6*	Within	2.89E-06	0.117
C12:0	8	BTB-00885512	24	30028775	*CHST9*	8314	6.08E-06	0.247
C10:0	13	BTB-01077939	26	7685110	***PRKG1***	Within	1.44E-05	0.586
C12:0	22	BTA-111275-no-rs	26	9195089	***MINPP1/PRKG1***	Within/851454	2.39E-05	0.969
C14:0	22	ARS-BFGL-NGS-113226	27	97306	*LOC100335608*	Within	1.34E-05	0.544
C12:0	15	BTB-01926888	27	16398882	*TRIML2/* ***ACSL1***	303782/2110549	1.34E-05	0.545
C12:0	5	BTB-01603522	27	16421445	*TRIML2/* ***ACSL1***	281219/2133112	3.55E-06	0.144
C12:0	18	ARS-BFGL-NGS-18922	29	21930571	*LUZP2*	1125590	1.72E-05	0.696
C14:0	24	ARS-BFGL-NGS-19057	29	44196154	*CDC42EP2*	1060	1.58E-05	0.640
C14:0	15	Hapmap60349-rs29021239	X	14062133	*ZNF280C*	Within	6.51E-06	0.264

Note:

a Rank represents ranking of significant SNPs within each oftrait; The *P*_value with bold type represents the significance of genome-wise level; The gene name with bold type represents the nearest known gene to the significant SNPs; The gene name with bold type and underline represents the nearest novel candidate gene to the significant SNPs.

**Table 3:** Genome-wise and suggestive significant SNPs for long-chain saturated fatty acid traits (LCFA).

Trait	Rank^b^	SNP	Chr.	Position(bp)	Nearest Gene/Candidate Gene	Distance(bp)	Raw P_value^a^	P_value Bonferroni
C16:0	1	ARS-BFGL-NGS-111692	0	0	*NA*	NA	1.84E-06	0.075
C22:0	1	ARS-BFGL-NGS-109692	1	15796320	*NCAM2*	432009	**6.70E-07**	**2.72E-02**
C18:0	78	ARS-BFGL-NGS-111491	1	68457522	*PTPLB*	6964	1.46E-05	0.593
C18:0	91	Hapmap50666-BTA-34589	1	68533156	*PTPLB*	Within	1.88E-05	0.761
C18:0	8	ARS-BFGL-NGS-76111	1	103219606	*SI*	14878	**7.51E-07**	**3.05E-02**
C18:0	25	BTB-00048807	1	106245603	*OTOL1*	454829	2.46E-06	0.100
C18:0	26	BTB-00048739	1	106295981	*OTOL1*	404451	2.77E-06	0.112
C18:0	16	ARS-BFGL-NGS-111111	1	146302724	*HSF2BP/* ***AGPAT3***	43214/402889	1.68E-06	0.068
C18:0	41	ARS-BFGL-NGS-109493	1	146354654	*HSF2BP/* ***AGPAT3***	Within/350959	5.28E-06	0.214
C18:0	42	BTA-56389-no-rs	1	146384457	*HSF2BP/* ***AGPAT3***	Within/321156	5.28E-06	0.214
C18:0	93	Hapmap59917-rs29012418	2	24519348	*METAP1D*	Within	1.92E-05	0.778
C18:0	88	ARS-BFGL-BAC-2793	2	47449015	*KIF5C*	Within	1.79E-05	0.726
C18:0	66	Hapmap53388-rs29010903	2	63581955	*MGAT5*	129790	1.18E-05	0.479
C18:0	63	BTB-01373917	2	79056259	*GYPC/* ***STAT1***	199326/837973	1.12E-05	0.453
C18:0	22	ARS-BFGL-NGS-33744	2	79388083	*GYPC/* ***STAT1***	83668/506149	2.26E-06	0.092
C18:0	52	ARS-BFGL-NGS-99030	2	98160191	*UNC80*	Within	9.14E-06	0.371
C18:0	11	ARS-BFGL-NGS-45691	2	128484790	*RUNX3/* ***FABP3***	144886/5700960	**9.56E-07**	**3.88E-02**
C18:0	30	ARS-BFGL-NGS-118924	2	128529102	*RUNX3/* ***FABP3***	100574/5745272	3.22E-06	0.131
C18:0	104	ARS-BFGL-NGS-58955	2	133620177	*HTR6*	Within	2.41E-05	0.978
C18:0	10	ARS-BFGL-NGS-45803	2	134246808	*IFFO2*	46005	**8.99E-07**	**3.65E-02**
C18:0	50	ARS-BFGL-NGS-15882	3	991777	*MPZL1*	Within	8.70E-06	0.353
C18:0	55	Hapmap34855-BES3_Contig373_1182	4	5549277	*IKZF1*	31842	1.00E-05	0.407
C18:0	79	BTA-122414-no-rs	4	34967013	*SEMA3D*	596525	1.50E-05	0.610
C16:0	12	Hapmap40292-BTA-71565	4	81400732	*C4H7orf10*	Within	1.39E-05	0.564
C18:0	7	Hapmap30257-BTA-142970	5	25358659	*USP44*	44459	**6.91E-07**	**2.81E-02**
C18:0	60	Hapmap41951-BTA-73168	5	28442563	*SLC4A8*	25682	1.05E-05	0.425
C18:0	73	Hapmap49848-BTA-106779	5	45089737	***CPM***	Within	1.31E-05	0.533
C18:0	12	Hapmap50366-BTA-46960	5	68610818	*CHST11*	Within	**1.02E-06**	**4.14E-02**
C16:0	3	Hapmap39862-BTA-74478	5	85672503	*BCAT1*	74712	4.88E-06	0.198
C16:0	7	BTB-00316348	7	64939808	*ATOX1*	Within	1.21E-05	0.490
C18:0	82	BTB-01553821	7	107940320	*EFNA5*	1105715	1.63E-05	0.663
C18:0	75	BTB-00995040	8	22411464	*MIR31*	123333	1.32E-05	0.536
C18:0	18	BTB-01709624	8	33060034	*LOC101904752*	1103805	1.86E-06	0.075
C18:0	43	BTA-99986-no-rs	8	33175156	*LOC101904752*	988683	5.54E-06	0.225
C18:0	61	BTB-01973796	8	34146856	*LOC101904752*	16983	1.07E-05	0.434
C18:0	24	BTB-01929442	8	34560427	*LOC101904752*	52452	2.45E-06	0.099
C18:0	70	ARS-BFGL-NGS-17346	8	70653923	*PEBP4*	Within	1.27E-05	0.514
C18:0	67	Hapmap57757-ss46526215	8	74949334	*BNIP3L*	Within	1.18E-05	0.481
C18:0	46	ARS-BFGL-NGS-105738	8	76089371	*APTX*	7156	6.77E-06	0.275
C18:0	53	BTB-00227581	8	76119002	*DNAJA1*	1365	9.23E-06	0.375
C18:0	29	BTB-00359112	8	76403422	*NFX1*	Within	3.14E-06	0.128
C18:0	28	ARS-BFGL-NGS-9052	8	78009328	*FRMD3*	181107	3.10E-06	0.126
C18:0	94	BTB-01900316	8	78226921	*UBQLN1*	Within	1.93E-05	0.783
C18:0	15	Hapmap23947-BTA-153013	8	78347212	*GKAP1*	Within	1.66E-06	0.067
C18:0	37	Hapmap54400-rs29020952	8	79534851	*NTRK2*	Within	4.82E-06	0.196
C18:0	76	Hapmap34874-BES3_Contig415_1312	8	79559282	*NTRK2*	Within	1.34E-05	0.545
C18:0	81	ARS-BFGL-NGS-101844	9	13292880	*SLC17A5*	12122	1.59E-05	0.644
C18:0	96	ARS-BFGL-NGS-101978	9	18123535	***HTR1B***	818548	2.04E-05	0.830
C18:0	74	ARS-BFGL-NGS-117379	9	97397264	*SOD2*	1895	1.32E-05	0.535
C18:0	45	ARS-BFGL-NGS-66090	10	11104820	*SERINC5*	Within	5.91E-06	0.240
C18:0	39	BTA-23031-no-rs	10	27606965	*OR4K13*	6376	4.93E-06	0.200
C16:0	5	ARS-BFGL-NGS-23492	10	34637715	*RASGRP1*	356723	1.13E-05	0.460
C16:0	10	ARS-BFGL-NGS-503	10	34705386	*RASGRP1*	424394	1.35E-05	0.547
C16:0	11	ARS-BFGL-NGS-4783	10	73487550	*SLC38A6*	35850	1.37E-05	0.555
C16:0	17	ARS-BFGL-NGS-14667	10	81481148	*GALNTL1*	Within	1.79E-05	0.728
C16:0	8	BTA-77299-no-rs	10	81549606	*SLC39A9*	Within	1.24E-05	0.503
C16:0	16	Hapmap44279-BTA-75297	10	84178157	*RGS6*	Within	1.75E-05	0.712
C18:0	102	BTB-00471219	11	32709462	*NRXN1*	Within	2.25E-05	0.915
C18:0	103	Hapmap25798-BTA-126388	11	32731961	*NRXN1*	Within	2.40E-05	0.976
C18:0	9	Hapmap51531-BTA-98947	11	57522675	*REG3A*	880289	**8.78E-07**	**3.57E-02**
C18:0	6	BTB-01328920	11	57639084	*LRRTM4*	792049	**5.79E-07**	**2.35E-02**
C18:0	40	BTA-33625-no-rs	11	58306286	*LRRTM4*	124847	5.07E-06	0.206
C18:0	27	Hapmap47549-BTA-25561	11	69506822	*LCLAT1*	108007	2.85E-06	0.116
C18:0	97	BTB-00866714	12	65771899	*LOC101907906*	250357	2.09E-05	0.850
C18:0	62	ARS-BFGL-BAC-5848	12	68657690	*GPC6*	Within	1.10E-05	0.446
C18:0	100	Hapmap51198-BTA-27036	12	68703184	*GPC6*	Within	2.15E-05	0.873
C18:0	1	ARS-BFGL-BAC-13788	12	69512332	*DCT*	Within	**9.17E-09**	**3.72E-04**
C18:0	65	Hapmap23511-BTA-119303	12	69768137	*SOX21*	24828	1.14E-05	0.463
C18:0	2	ARS-BFGL-NGS-45730	12	70260457	*ABCC4*	51353	**1.05E-08**	**4.24E-04**
C18:0	101	ARS-BFGL-NGS-40278	13	67122063	*BLCAP/* ***ACSS2***	Within/2280543	2.25E-05	0.914
C16:0	4	ARS-BFGL-NGS-4939	14	1801116	***DGAT1***	Within	9.04E-06	0.367
C18:0	57	BTB-00557532	14	24643266	*XKR4*	28554	1.03E-05	0.416
C18:0	64	ARS-BFGL-NGS-110022	14	40616098	*CRISPLD1*	144633	1.12E-05	0.456
C18:0	36	ARS-BFGL-NGS-2025	14	65452336	*ZNF706*	103905	4.72E-06	0.192
C18:0	19	Hapmap35102-BES3_Contig324_378	14	66523007	*RGS22*	Within	1.87E-06	0.076
C18:0	17	ARS-BFGL-BAC-1991	14	82648300	*SNX16*	491346	1.73E-06	0.070
C18:0	34	ARS-BFGL-NGS-12066	15	27291268	*BUD13*	542743	3.98E-06	0.162
C18:0	54	BTB-01465034	15	49987359	*LOC784376*	1153	9.65E-06	0.392
C16:0	2	ARS-BFGL-NGS-97658	15	68069900	*C15H11orf74*	158748	2.72E-06	0.110
C18:0	92	BTA-37923-no-rs	15	85011763	*GLB1L2*	Within	1.88E-05	0.761
C18:0	3	ARS-BFGL-NGS-68533	16	60615012	*LOC101902340*	35998	**2.71E-07**	**1.10E-02**
C18:0	31	ARS-BFGL-NGS-102798	16	61477887	*RALGPS2*	82055	3.31E-06	0.134
C18:0	95	BTB-00653808	16	62179726	*AXDND1*	Within	2.01E-05	0.817
C18:0	87	ARS-BFGL-NGS-117800	16	62871926	*LHX4*	Within	1.75E-05	0.711
C18:0	83	ARS-BFGL-NGS-102835	17	3855180	*SFRP2*	17042	1.67E-05	0.678
C18:0	69	ARS-BFGL-NGS-100229	17	4153159	*KIAA0922*	Within	1.21E-05	0.493
C18:0	14	ARS-BFGL-NGS-77485	17	4926550	*TMEM154*	39267	1.47E-06	0.059
C18:0	38	ARS-BFGL-NGS-118611	17	5357573	*FBXW7*	23715	4.91E-06	0.199
C18:0	89	ARS-BFGL-NGS-38059	17	5829384	*PET112*	151784	1.79E-05	0.728
C18:0	84	ARS-BFGL-NGS-114953	17	9753430	*NR3C2*	Within	1.70E-05	0.688
C18:0	98	ARS-BFGL-NGS-111098	17	67193210	*PIWIL3*	27876	2.10E-05	0.851
C18:0	13	ARS-BFGL-NGS-71116	17	68002540	*MYO18B*	Within	**1.09E-06**	**4.41E-02**
C18:0	86	ARS-BFGL-NGS-75816	19	36674728	*ANKRD40/* ***SREBF1***	Within/1424056	1.70E-05	0.692
C18:0	71	BTB-01790846	20	13263157	*SREK1*	128568	1.27E-05	0.516
C18:0	99	Hapmap56230-rs29025779	20	25535571	*NDUFS4*	12189	2.14E-05	0.869
C18:0	105	ARS-BFGL-NGS-118998	20	32030332	***GHR*** */* ***OXCT1***	Within/653664	2.43E-05	0.988
C18:0	48	ARS-BFGL-NGS-87102	20	49859323	*CDH12*	585652	7.71E-06	0.313
C18:0	23	BTB-01475042	20	51845309	*CDH12*	220958	2.29E-06	0.093
C18:0	20	ARS-BFGL-NGS-51112	20	52816141	*CDH18*	593735	1.89E-06	0.077
C16:0	18	BTB-00818669	21	40502782	*PRKD1*	25243	1.95E-05	0.790
C16:0	13	BTB-01240884	21	41484660	*G2E3*	128084	1.48E-05	0.600
C16:0	9	Hapmap24313-BTA-29957	21	51294895	*LRFN5*	476940	1.30E-05	0.526
C18:0	58	ARS-BFGL-BAC-46707	23	5756655	*TINAG*	196853	1.03E-05	0.419
C16:0	15	BTA-56520-no-rs	23	32581285	*LOC537017/* ***BTN1A1***	59797/1214677	1.73E-05	0.700
C18:0	33	Hapmap58547-rs29023020	23	35911017	*LOC100847951/* ***PRL***	Within/797267	3.81E-06	0.155
C18:0	49	BTA-58814-no-rs	24	5828401	*CBLN2*	207345	8.20E-06	0.333
C18:0	80	BTB-00878928	24	5883369	*CBLN2*	262313	1.51E-05	0.612
C18:0	59	Hapmap34424-BES10_Contig566_926	24	6408329	*CBLN2*	787273	1.04E-05	0.421
C18:0	4	ARS-BFGL-NGS-109955	24	11625175	*CDH7*	411164	**3.29E-07**	**1.33E-02**
C18:0	51	BTA-24495-no-rs	24	15527942	*PIK3C3*	1180676	8.82E-06	0.358
C18:0	56	ARS-BFGL-NGS-13314	24	45775076	*SLC14A2*	Within	1.01E-05	0.408
C18:0	35	BTA-59652-no-rs	25	19315456	*LOC524391*	Within	4.37E-06	0.178
C16:0	6	BTB-01619101	26	16035322	*CYP2C87*	Within	1.16E-05	0.472
C18:0	72	ARS-BFGL-NGS-12828	26	37018466	*GFRA1*	Within	1.29E-05	0.524
C18:0	21	ARS-BFGL-NGS-100468	26	38060272	*PDZD8*	39152	2.10E-06	0.085
C18:0	47	ARS-BFGL-NGS-111901	26	41183634	*WDR11*	28744	7.62E-06	0.309
C18:0	68	UA-IFASA-5698	26	42673967	*HTRA1*	Within	1.21E-05	0.491
C18:0	77	Hapmap60810-rs29012623	27	12355411	*LOC100848735*	130088	1.39E-05	0.565
C20:0	2	BTB-00965197	27	26813022	*NRG1*	Within	1.93E-05	0.783
C20:0	1	ARS-BFGL-NGS-77002	27	30883081	*UNC5D*	Within	3.76E-06	0.153
C18:0	85	Hapmap35611-SCAFFOLD120249_17244	27	44005137	*ZNF385D*	5580	1.70E-05	0.691
C16:0	14	Hapmap27418-BTA-147969	29	23469370	*LOC540991*	19676	1.66E-05	0.675
C18:0	32	ARS-BFGL-NGS-19057	29	44196154	*CDC42EP2*	1060	3.65E-06	0.148
C18:0	5	ARS-BFGL-NGS-11681	X	3622615	*Gene desert*	NA	**3.74E-07**	**1.52E-02**
C18:0	44	Hapmap48540-BTA-97806	X	8700029	*ODZ1*	Within	5.77E-06	0.234
C18:0	90	BTB-01492502	X	8724630	*ODZ1*	Within	1.84E-05	0.746

Note: see note to Table 2.

**Table 4:** Genome-wise and suggestive significant SNPs for monounsaturated and polyunsaturated fatty acid traits (MUFA and PUFA).

Trait	Rank^b^	SNP	Chr.	Position(bp)	Nearest Gene/Candidate Gene	Distance(bp)	Raw P_value^a^	P_value Bonferroni
C14:1	34	ARS-BFGL-NGS-15914	0	0	*NA*	NA	**1.20E-06**	**4.87E-02**
CLA	2	ARS-BFGL-NGS-43953	1	113855984	*GPR149*	288255	1.31E-05	0.531
C14:1	39	ARS-BFGL-BAC-42858	2	33401608	*LOC534542*	231071	2.02E-06	0.082
CLA	4	BTB-01649999	3	48062099	*RWDD3*	336059	1.65E-05	0.672
C18:1n9c	9	Hapmap42304-BTA-73062	5	5049476	*KRR1/* ***OSBPL8***	14567/770655	1.25E-05	0.509
C18:1n9c	19	ARS-BFGL-NGS-8796	5	29095603	*LOC510716*	Within	2.25E-05	0.915
C18:1n9c	8	ARS-BFGL-NGS-69056	5	42285835	*CPNE8*	135184	1.18E-05	0.479
C18:1n9c	10	Hapmap39862-BTA-74478	5	85672503	*BCAT1*	74712	1.27E-05	0.515
C18:1n9c	6	ARS-BFGL-NGS-99256	5	104714350	*VWF/* ***OLR1***	Within/4458707	8.88E-06	0.361
C14:1	64	ARS-BFGL-NGS-110361	7	15782979	*COL5A3*	Within	2.03E-05	0.826
C14:1	61	ARS-BFGL-NGS-104050	7	62839580	*CSNK1A1*	13832	1.48E-05	0.600
C14:1	65	Hapmap50476-BTA-79543	7	62889548	*CSNK1A1*	Within	2.17E-05	0.882
C18:1n9c	18	BTB-00316291	7	64892251	*SPARC*	Within	2.15E-05	0.874
C18:1n9c	12	BTB-00316348	7	64939808	*ATOX1*	5940	1.32E-05	0.534
CLA	6	BTB-01541157	7	84582299	*ATP6AP1L*	15187	1.78E-05	0.722
C18:2n6c	5	BTA-19330-no-rs	7	93289032	*ARRDC3*	35938	9.00E-06	0.366
CLA	5	Hapmap27874-BTA-146513	9	53871363	*GPR63*	53530	1.66E-05	0.676
C18:1n9c	15	Hapmap50126-BTA-83733	9	55449737	*LOC101907134*	18763	1.49E-05	0.606
C16:1	6	ARS-BFGL-NGS-115094	9	93183596	*TIAM2*	Within	1.88E-05	0.762
C16:1	4	ARS-BFGL-NGS-57866	9	93246183	*TFB1M/TIAM2*	Within	1.34E-05	0.545
C18:1n9c	7	BTB-01332998	10	73466092	*SLC38A6*	14392	1.02E-05	0.414
C18:1n9c	5	ARS-BFGL-NGS-4783	10	73487550	*SLC38A6*	35850	5.75E-06	0.233
C16:1	5	Hapmap38187-BTA-105082	12	69216840	*DCT*	286101	1.51E-05	0.614
C16:1	2	Hapmap53988-rs29024591	13	65855988	*EPB41L1/* ***ACSS2***	23900/1014468	9.31E-06	0.378
C16:1	3	BPI-2	13	67833153	*LOC514211/* ***ACSS2***	Within/2991633	1.16E-05	0.470
C18:2n6c	4	ARS-BFGL-NGS-57820	14	1651311	*LOC100294916/* ***DGAT1***	Within/144114	2.47E-06	0.100
C14:1	60	ARS-BFGL-NGS-4939	14	1801116	***DGAT1***	Within	1.45E-05	0.590
C18:1n9c	2	ARS-BFGL-NGS-4939	14	1801116	***DGAT1***	Within	3.01E-06	0.122
C18:2n6c	1	ARS-BFGL-NGS-4939	14	1801116	***DGAT1***	Within	**6.87E-08**	**2.79E-03**
C14:1	25	Hapmap52798-ss46526455	14	1923292	*MAF1/* ***DGAT1***	Within/118454	**2.32E-07**	**9.42E-03**
C18:2n6c	3	ARS-BFGL-NGS-107379	14	2054457	*LOC786966/* ***DGAT1***	460/249619	**1.13E-06**	**4.57E-02**
C18:1n9c	1	ARS-BFGL-NGS-100480	14	4364952	*TRAPPC9*	Within	1.95E-06	0.079
C14:1	43	Hapmap32234-BTC-048199	14	7314869	*KHDRBS3*	117233	3.48E-06	0.141
C18:2n6c	2	ARS-BFGL-NGS-114448	16	32095335	*SMYD3*	Within	**4.72E-07**	**1.92E-02**
C16:1	1	BTB-01090859	16	63248646	*XPR1*	51157	4.29E-06	0.174
C14:1	67	ARS-BFGL-NGS-66923	17	18910914	*CCRN4L*	Within	2.36E-05	0.958
C14:1	37	ARS-BFGL-NGS-102933	17	19315294	*CCRN4L*	404368	1.46E-06	0.059
C14:1	16	BTA-91575-no-rs	17	20099837	*SLC7A11*	291555	**3.42E-08**	**1.39E-03**
C14:1	47	ARS-BFGL-NGS-25840	17	23694684	*LOC783956*	163671	4.48E-06	0.182
C14:1	32	BTB-01585209	17	44910178	*ZNF605*	53460	**9.81E-07**	**3.98E-02**
C14:1	17	ARS-BFGL-NGS-109854	17	44963124	*ZNF605*	514	**4.23E-08**	**1.72E-03**
C18:1n9c	16	ARS-BFGL-NGS-87368	19	7762820	*C19H17orf67*	67914	1.76E-05	0.713
C14:1	53	BTA-117074-no-rs	20	247319	*PANK3*	42534	7.72E-06	0.313
C14:1	62	ARS-BFGL-NGS-114602	20	1052840	*SLIT3*	979	1.52E-05	0.618
C14:1	57	ARS-BFGL-NGS-101925	20	1202954	*SLIT3*	151093	8.96E-06	0.364
C18:1n9c	4	ARS-BFGL-NGS-116806	20	36450009	*GDNF*	180881	4.66E-06	0.189
C18:1n9c	13	ARS-BFGL-NGS-14031	20	36561330	*GDNF*	69560	1.46E-05	0.594
C18:1n9c	11	ARS-USMARC-Parent-DQ990835-rs29012811	20	36570529	*GDNF*	60361	1.28E-05	0.519
CLA	1	BTA-50525-no-rs	20	36917645	*WDR70*	Within	1.16E-05	0.469
C18:1n9c	20	ARS-BFGL-NGS-111420	20	37553988	*SLC1A3*	42121	2.30E-05	0.935
C18:1n9c	17	Hapmap57276-ss46526009	20	37596667	*SLC1A3*	Within	2.04E-05	0.829
C18:2n6c	6	BTB-01340958	20	60400902	*DNAH5*	840190	1.70E-05	0.689
CLA	7	Hapmap26512-BTA-52638	21	55483469	*MIS18BP1*	175	1.85E-05	0.752
C14:1	54	ARS-BFGL-BAC-46707	23	5756655	*TINAG*	196853	7.82E-06	0.318
CLA	3	BTA-24495-no-rs	24	15527942	*LOC783699*	51814	1.57E-05	0.637
C18:1n9c	3	ARS-BFGL-BAC-28144	25	2606575	*LOC788915*	21466	4.34E-06	0.176
C14:1	48	Hapmap54064-rs29011996	26	5526925	*PCDH15*	Within	4.58E-06	0.186
C14:1	35	ARS-BFGL-NGS-13746	26	9866940	*RNLS*	65137	1.42E-06	0.058
C14:1	59	BTA-61921-no-rs	26	10255258	***LIPJ***	7406	1.42E-05	0.577
C14:1	21	ARS-BFGL-NGS-21794	26	10397362	*LOC100336557/* ***LIPK***	16309/35288	**9.57E-08**	**3.88E-03**
C14:1	51	BTA-08775-rs29022332	26	11201198	*SLC16A12*	39720	6.64E-06	0.269
C14:1	44	ARS-BFGL-NGS-53115	26	11528933	*KIF20B*	61222	3.82E-06	0.155
C14:1	24	ARS-BFGL-NGS-63853	26	11942868	*MIR2895*	67662	**2.07E-07**	**8.42E-03**
C14:1	36	ARS-BFGL-NGS-12381	26	12200948	*LOC100141242*	24477	1.45E-06	0.059
C14:1	20	BTB-01908417	26	12268427	*LOC784522*	15486	**5.62E-08**	**2.28E-03**
C14:1	66	ARS-BFGL-NGS-41148	26	12364119	*HTR7*	10881	2.20E-05	0.892
C14:1	28	Hapmap52817-rs29011969	26	14155229	*HHEX*	29160	**3.97E-07**	**1.61E-02**
C14:1	38	ARS-BFGL-NGS-110475	26	15604631	*PLCE1*	Within	1.54E-06	0.063
C14:1	19	ARS-BFGL-NGS-29299	26	16614068	*PDLIM1/* ***SORBS1***	Within/73061	**5.42E-08**	**2.20E-03**
C14:1	23	Hapmap41595-BTA-60800	26	16791783	***SORBS1***	Within	**2.07E-07**	**8.41E-03**
C14:1	40	Hapmap58930-rs29010490	26	16822073	***SORBS1***	Within	2.34E-06	0.095
C14:1	42	ARS-BFGL-NGS-41056	26	18906121	*CRTAC1*	Within	2.70E-06	0.110
C14:1	45	ARS-BFGL-NGS-116902	26	18967997	*CRTAC1*	Within	4.31E-06	0.175
C14:1	55	ARS-BFGL-NGS-25126	26	18994785	*CRTAC1*	Within	7.84E-06	0.318
C14:1	15	ARS-BFGL-NGS-23064	26	20365711	*NKX2-3/* ***SCD1***	34516/767033	**2.93E-08**	**1.19E-03**
C14:1	14	ARS-BFGL-NGS-77668	26	20393457	*NKX2-3/* ***SCD1***	6770/744488	**2.23E-08**	**9.05E-04**
C14:1	33	BTB-00930925	26	20474308	*SLC25A28/* ***SCD1***	Within/658436	**1.08E-06**	**4.39E-02**
C14:1	22	ARS-BFGL-NGS-39397	26	20716721	*DNMBP/* ***SCD1***	Within/416023	**1.37E-07**	**5.55E-03**
C14:1	27	BTB-00930720	26	20903573	*LOC511498/* ***SCD1***	Within/244744	**2.86E-07**	**1.16E-02**
C14:1	56	Hapmap46411-BTA-15820	26	20984335	***CHUK*** */* ***SCD1***	Within/148409	8.29E-06	0.336
C14:1	31	Hapmap31825-BTA-158647	26	21056547	*PKD2L1/* ***SCD1***	Within/76197	**5.10E-07**	**2.07E-02**
C14:1	11	Hapmap33073-BTA-162864	26	21180893	***SCD1***	32576	**1.31E-08**	**5.33E-04**
C14:1	1	BTB-00931481	26	21226405	*WNT8B/* ***SCD1***	14100/78088	**7.08E-13**	**2.87E-08**
C14:1	12	ARS-BFGL-NGS-110077	26	21322557	*HIF1AN/* ***SCD1***	22399/174240	**1.54E-08**	**6.23E-04**
C14:1	10	ARS-BFGL-NGS-108305	26	21363670	*HIF1AN/* ***SCD1***	63512/215353	**8.07E-09**	**3.28E-04**
C14:1	9	BTB-00931586	26	21409429	*PAX2/* ***SCD1***	61334/261112	**2.25E-09**	**9.12E-05**
C14:1	5	ARS-BFGL-NGS-114149	26	21702714	*LZTS2/* ***SCD1***	656/564769	**5.50E-10**	**2.23E-05**
C14:1	6	ARS-BFGL-NGS-116481	26	21977581	*LOC100847491/* ***SCD1***	10062/829264	**5.77E-10**	**2.34E-05**
C14:1	7	Hapmap24832-BTA-138805	26	22016380	*BTRC/* ***SCD1***	Within/868063	**7.18E-10**	**2.92E-05**
C14:1	8	ARS-BFGL-NGS-6259	26	22059103	*BTRC/* ***SCD1***	Within/910786	**7.23E-10**	**2.93E-05**
C14:1	4	BTB-00932332	26	22118554	*BTRC/* ***SCD1***	Within/970237	**3.56E-10**	**1.45E-05**
C14:1	3	ARS-BFGL-NGS-107403	26	22889812	***NFKB2***	1586	**2.22E-12**	**9.02E-08**
C14:1	18	Hapmap48222-BTA-122240	26	23641881	*C26H10orf26*	Within	**4.53E-08**	**1.84E-03**
C14:1	29	Hapmap49372-BTA-91009	26	23689229	*C26H10orf26*	3017	**4.66E-07**	**1.89E-02**
C14:1	46	BTA-60918-no-rs	26	23853334	*CNNM2*	Within	4.40E-06	0.179
C14:1	13	BTA-60935-no-rs	26	23876476	*CNNM2*	Within	**1.56E-08**	**6.33E-04**
C14:1	52	ARS-BFGL-NGS-2180	26	24477962	*SH3PXD2A*	Within	7.46E-06	0.303
C14:1	49	ARS-BFGL-NGS-1092	26	24531763	*SH3PXD2A*	Within	4.90E-06	0.199
C14:1	2	ARS-BFGL-NGS-118189	26	24786731	*SLK*	Within	**1.69E-12**	**6.87E-08**
C14:1	58	UA-IFASA-4715	26	25314352	*CCDC147*	27285	9.16E-06	0.372
C14:1	30	Hapmap28763-BTA-162328	26	26757136	*SORCS3/* ***ECHS1***	358845/891528	**5.05E-07**	**2.05E-02**
C14:1	26	BTA-87355-no-rs	26	27251857	*SORCS1*	558341	**2.59E-07**	**1.05E-02**
C14:1	41	BTA-10873-rs29016424	28	18317414	*RTKN2*	41359	2.35E-06	0.095
C14:1	63	ARS-BFGL-NGS-118293	28	45409471	*CXCL12*	Within	1.77E-05	0.718
C18:1n9c	14	BTB-01088519	29	22161137	*CCDC179*	85675	1.49E-05	0.605
C14:1	50	ARS-BFGL-NGS-27560	X	146596721	*STS*	Within	5.19E-06	0.211

Note: see note to Table 2.

**Table 5:** Genome-wise and suggestive significant SNPs for indices of fatty acid traits.

Trait	Rank^b^	SNP	Chr.	Position(bp)	Nearest Gene/Candidate Gene	Distance(bp)	Raw P_value^a^	P_value Bonferroni
C14index	26	ARS-BFGL-NGS-15914	0	0	*NA*	NA	**2.59E-09**	**1.05E-04**
C14index	38	UA-IFASA-5862	0	0	*NA*	NA	**8.04E-08**	**3.26E-03**
C18index	65	BTB-00270136	0	0	*NA*	NA	1.19E-05	0.482
C18index	91	Hapmap60647-rs29027341	0	0	*NA*	NA	2.25E-05	0.912
C18index	23	BTA-111771-no-rs	1	37174447	*EPHA3*	50365	2.86E-06	0.116
C18index	22	ARS-BFGL-NGS-106725	1	41577955	*EPHA6*	Within	2.74E-06	0.111
C18index	45	ARS-BFGL-NGS-115763	1	66139653	*GTF2E1*	67771	6.14E-06	0.249
C18index	35	BTB-00032200	1	67764428	*DIRC2*	Within	3.83E-06	0.156
C18index	33	ARS-BFGL-NGS-35839	1	102589009	*BCHE*	178966	3.69E-06	0.150
C18index	24	ARS-BFGL-NGS-111111	1	146302724	*HSF2BP/* ***AGPAT3***	43214/402889	2.92E-06	0.118
C18index	12	ARS-BFGL-NGS-109493	1	146354654	*HSF2BP/* ***AGPAT3***	Within/350959	**1.13E-06**	**4.59E-02**
C18index	13	BTA-56389-no-rs	1	146384457	*HSF2BP/* ***AGPAT3***	Within/321156	**1.13E-06**	**4.59E-02**
C18index	32	ARS-BFGL-NGS-76347	1	146704618	***AGPAT3***	995	3.66E-06	0.149
C18index	84	Hapmap59917-rs29012418	2	24519348	*METAP1D*	Within	1.90E-05	0.771
C18index	21	Hapmap53388-rs29010903	2	63581955	*MGAT5*	129790	2.65E-06	0.107
C18index	3	ARS-BFGL-NGS-33744	2	79388083	*GYPC/* ***STAT1***	83687/506149	**8.95E-08**	**3.63E-03**
C18index	31	Hapmap53419-rs29015159	2	88205436	*SATB2*	43199	3.57E-06	0.145
C18index	5	Hapmap33966-BES2_Contig368_774	2	88545567	*SATB2*	90018	**2.56E-07**	**1.04E-02**
C18index	50	ARS-BFGL-NGS-23872	2	95199905	*ADAM23*	Within	7.34E-06	0.298
C18index	76	ARS-BFGL-NGS-98354	2	95512347	*FASTKD2*	Within	1.47E-05	0.598
C18index	55	ARS-BFGL-NGS-99030	2	98160191	*UNC80*	Within	8.85E-06	0.359
C18index	15	ARS-BFGL-NGS-45691	2	128484790	*RUNX3/* ***FABP3***	144886/5700960	1.41E-06	0.057
C18index	9	ARS-BFGL-NGS-118924	2	128529102	*RUNX3/* ***FABP3***	100574/5745272	**6.04E-07**	**2.45E-02**
C18index	28	Hapmap30257-BTA-142970	5	25358659	*USP44*	44459	3.24E-06	0.131
C18index	75	ARS-BFGL-NGS-38038	5	27992179	*NR4A1*	Within	1.45E-05	0.588
C18index	59	Hapmap41951-BTA-73168	5	28442563	*SLC4A8*	25682	9.43E-06	0.383
C18index	16	ARS-BFGL-NGS-8796	5	29095603	*LOC510716*	Within	1.82E-06	0.074
C18index	92	ARS-BFGL-NGS-53488	5	41154328	*PRICKLE1*	166915	2.35E-05	0.953
C18index	47	ARS-BFGL-NGS-116897	5	95743746	*PLBD1/* ***OLR1***	Within/4500525	6.49E-06	0.264
C18index	73	BTB-01685239	6	12282881	*UGT8*	81408	1.44E-05	0.586
C18index	14	BTB-00246150	6	20993424	*PPA2*	Within	**1.15E-06**	**4.68E-02**
C18index	30	Hapmap26001-BTC-038813	6	44926243	***PPARGC1A***	Within	3.54E-06	0.144
C18index	62	Hapmap31284-BTC-039204	6	45096462	***PPARGC1A***	135929	1.09E-05	0.443
C18index	87	Hapmap49746-BTA-76106	6	46140090	*LGI2/* ***PPARGC1A***	Within/82649	1.97E-05	0.801
C14index	52	ARS-BFGL-NGS-106015	6	61199572	*RBM47*	Within	2.03E-06	0.082
C14index	54	ARS-BFGL-NGS-80548	7	6434821	*C7H19orf44*	Within	2.79E-06	0.113
C14index	42	ARS-BFGL-NGS-110361	7	15782979	*COL5A3*	Within	**5.50E-07**	**2.23E-02**
C14index	78	ARS-BFGL-NGS-104050	7	62839580	*CSNK1A1*	13832	1.70E-05	0.691
C18index	77	BTB-00316650	7	65098028	*GLRA1*	Within	1.67E-05	0.679
C18index	63	BTB-01687547	8	20989026	*LOC101905651*	328218	1.13E-05	0.458
C18index	48	ARS-BFGL-NGS-9052	8	78009328	*FRMD3*	181107	6.84E-06	0.278
C18index	90	ARS-BFGL-NGS-106379	8	113159018	*TSSC1*	90395	2.24E-05	0.909
C18index	52	ARS-BFGL-NGS-15823	9	28887462	*PKIB*	Within	8.15E-06	0.331
C18index	10	BTB-01332998	10	73466092	*SLC38A6*	15901	**6.76E-07**	**2.74E-02**
C18index	29	ARS-BFGL-NGS-4783	10	73487550	*SLC38A6*	37359	3.30E-06	0.134
C18index	78	ARS-BFGL-NGS-22113	10	73551579	*TMEM30B*	94651	1.68E-05	0.683
C18index	26	BTB-00471219	11	32709462	*NRXN1*	Within	3.14E-06	0.128
C18index	44	Hapmap25798-BTA-126388	11	32731961	*NRXN1*	Within	6.07E-06	0.247
C18index	6	Hapmap40257-BTA-91916	11	32789048	*NRXN1*	Within	**3.25E-07**	**1.32E-02**
C18index	93	ARS-BFGL-NGS-85007	11	53430229	*CTNNA2*	1292050	2.37E-05	0.964
C18index	72	BTB-01079278	11	57078447	*REG3A*	436061	1.39E-05	0.563
C18index	60	BTB-01079350	11	57107070	*REG3A*	464684	9.51E-06	0.386
C18index	1	Hapmap51531-BTA-98947	11	57522675	*REG3A*	880289	**2.08E-08**	**8.44E-04**
C18index	2	BTB-01328920	11	57639084	*LRRTM4*	792049	**4.34E-08**	**1.76E-03**
C18index	54	ARS-BFGL-NGS-114087	11	64057850	*SPRED2*	293893	8.21E-06	0.333
C18index	25	ARS-BFGL-NGS-43985	11	64184454	*SPRED2*	420497	3.00E-06	0.122
C18index	40	ARS-BFGL-NGS-91014	11	65493222	*C11H2orf66*	Within	4.88E-06	0.198
C16index	8	ARS-BFGL-NGS-110868	11	75329413	*KLHL29*	Within	1.65E-05	0.670
C18index	89	UA-IFASA-2295	11	97754180	*RALGPS1*	Within	2.16E-05	0.878
C18index	27	BTA-119672-no-rs	11	102911946	*AK8*	Within	3.21E-06	0.130
C18index	71	BTB-00490466	12	47836570	*DIS3*	Within	1.37E-05	0.557
C14index	81	Hapmap42477-BTA-22799	12	48645720	*KLF12*	181742	2.25E-05	0.914
C18index	83	Hapmap57649-rs29022414	12	66068259	*GPC5*	223887	1.85E-05	0.753
C16index	2	Hapmap38187-BTA-105082	12	69216840	*DCT*	286101	7.38E-06	0.300
C18index	4	ARS-BFGL-BAC-13788	12	69512332	*DCT*	Within	**1.79E-07**	**7.27E-03**
C18index	46	ARS-BFGL-NGS-45730	12	70260457	*ABCC4*	49496	6.28E-06	0.255
C14index	55	ARS-BFGL-NGS-13252	12	81184160	*GGACT*	25567	3.04E-06	0.123
C16index	6	Hapmap53988-rs29024591	13	65855988	*EPB41L1/* ***ACSS2***	23900/1014468	1.19E-05	0.482
C16index	9	Hapmap40712-BTA-33406	13	67101174	*BLCAP/* ***ACSS2***	13538/2259654	1.74E-05	0.705
C18index	61	ARS-BFGL-NGS-40278	13	67122063	*BLCAP/* ***ACSS2***	Within/2280543	9.81E-06	0.398
C16index	3	BPI-2	13	67833153	*LOC514211/* ***ACSS2***	Within/2991633	7.51E-06	0.305
C16index	10	ARS-BFGL-NGS-107113	13	67958189	*LOC514978/* ***ACSS2***	Within/3116669	1.83E-05	0.743
C16index	1	Hapmap55254-rs29014939	13	69042143	*DHX35*	573959	5.20E-06	0.211
C18index	41	Hapmap30381-BTC-005750	14	1463676	*C14H8orf33/* ***DGAT1***	23690/331749	5.62E-06	0.228
C14index	73	ARS-BFGL-NGS-57820	14	1651311	*LOC100294916/* ***DGAT1***	Within/144114	1.03E-05	0.419
C14index	75	ARS-BFGL-NGS-4939	14	1801116	***DGAT1***	Within	1.15E-05	0.469
C18index	88	ARS-BFGL-NGS-4939	14	1801116	***DGAT1***	Within	2.00E-05	0.812
C14index	47	Hapmap52798-ss46526455	14	1923292	*MAF1/* ***DGAT1***	Within/118454	**1.08E-06**	**4.39E-02**
C14index	80	Hapmap30986-BTC-056068	14	10346734	*EFR3A*	64293	1.97E-05	0.799
C18index	51	BTB-00557532	14	24643266	*XKR4*	28554	7.35E-06	0.299
C18index	82	ARS-BFGL-BAC-10245	14	31819743	*PDE7A*	Within	1.80E-05	0.731
C18index	34	ARS-BFGL-NGS-2025	14	65452336	*ZNF706*	103905	3.79E-06	0.154
C18index	8	Hapmap35102-BES3_Contig324_378	14	66523007	*RGS22*	Within	**5.63E-07**	**2.29E-02**
C18index	58	UA-IFASA-4785	14	71096693	*C14H8orf37*	300038	9.36E-06	0.380
C18index	79	BTB-01296218	15	17344116	*ALKBH8*	21050	1.77E-05	0.720
C18index	39	ARS-BFGL-NGS-35704	15	18335423	*C15H11orf65*	Within	4.83E-06	0.196
C18index	37	ARS-BFGL-NGS-12066	15	27291268	*BUD13*	542743	4.33E-06	0.176
C18index	43	BTA-36518-no-rs	15	32983903	*SORL1*	228564	6.04E-06	0.245
C18index	81	Hapmap56991-rs29010083	15	81595546	*LOC538839*	52784	1.78E-05	0.724
C18index	68	Hapmap46697-BTA-38171	16	2899256	*NUAK2*	Within	1.24E-05	0.501
C18index	7	ARS-BFGL-NGS-68533	16	60615012	*LOC101902340*	35998	**5.39E-07**	**2.19E-02**
C16index	7	BTB-01090859	16	63248646	*XPR1*	51157	1.51E-05	0.615
C18index	64	ARS-BFGL-NGS-36880	16	73736551	*SLC30A1*	28425	1.14E-05	0.465
C14index	83	ARS-BFGL-NGS-38696	17	18398611	*MGST2*	29230	2.29E-05	0.929
C14index	64	ARS-BFGL-NGS-66923	17	18910914	*CCRN4L*	Within	5.55E-06	0.225
C14index	50	ARS-BFGL-NGS-102933	17	19315294	*CCRN4L*	404368	1.81E-06	0.074
C14index	39	BTA-91575-no-rs	17	20099837	*SLC7A11*	291555	**2.96E-07**	**1.20E-02**
C14index	79	Hapmap51443-BTA-40619	17	20996847	*PCDH18*	380577	1.72E-05	0.698
C14index	53	BTB-01585209	17	44910178	*ZNF605*	53460	2.79E-06	0.113
C14index	35	ARS-BFGL-NGS-109854	17	44963124	*ZNF605*	514	**6.46E-08**	**2.62E-03**
C18index	19	ARS-BFGL-NGS-71116	17	68002540	*MYO18B*	Within	2.41E-06	0.098
C18index	36	ARS-BFGL-NGS-37725	17	68490453	*TPST2*	Within	4.30E-06	0.175
C18index	74	BTB-01790846	20	13263157	*SREK1*	128568	1.44E-05	0.586
C18index	85	ARS-BFGL-BAC-2469	20	33433160	*HEATR7B2/* ***OXCT1***	22507/584434	1.93E-05	0.784
C18index	56	ARS-BFGL-NGS-76756	20	33491273	*HEATR7B2/* ***OXCT1***	Within/642547	9.20E-06	0.373
C18index	49	BTB-01423653	20	38578200	*SPEF2/* ***PRLR***	Within/495046	6.96E-06	0.282
C18index	42	BTB-01423676	20	38606353	*SPEF2/* ***PRLR***	Within/466893	6.02E-06	0.244
C18index	20	Hapmap30570-BTA-152778	20	38761711	*SPEF2/* ***PRLR***	154961/311535	2.55E-06	0.104
C18index	80	ARS-BFGL-NGS-99716	21	63560239	*VRK1*	216337	1.78E-05	0.723
C18index	53	ARS-BFGL-NGS-39459	22	50474049	*CACNA2D2*	Within	8.15E-06	0.331
C18index	69	Hapmap54558-rs29009598	24	29187804	*CDH2*	Within	1.27E-05	0.517
C16index	4	ARS-BFGL-NGS-45679	24	42582505	*APCDD1*	Within	7.82E-06	0.318
C16index	5	Hapmap31260-BTC-015327	25	2224930	*ZG16B*	Within	8.51E-06	0.346
C14index	51	Hapmap54064-rs29011996	26	5526925	*PCDH15*	Within	2.02E-06	0.082
C14index	77	BTB-01077939	26	7685110	***PRKG1***	Within	1.35E-05	0.548
C14index	27	ARS-BFGL-NGS-13746	26	9866940	*RNLS*	65137	**3.71E-09**	**1.51E-04**
C14index	72	Hapmap58185-rs29022254	26	10002077	*RNLS*	Within	1.02E-05	0.415
C14index	36	BTA-61921-no-rs	26	10255258	***LIPJ***	7406	**6.99E-08**	**2.84E-03**
C14index	20	ARS-BFGL-NGS-21794	26	10397362	*LOC100336557/* ***LIPK***	16309/35288	**4.20E-10**	**1.70E-05**
C14index	68	Hapmap59335-rs29016866	26	10689379	*ACTA2*	9731	8.36E-06	0.339
C14index	76	BTA-111857-no-rs	26	10815586	*FAS*	67560	1.17E-05	0.476
C14index	56	BTB-00924013	26	10922061	*CH25H*	54132	3.05E-06	0.124
C14index	48	BTA-08775-rs29022332	26	11201198	*SLC16A12*	39720	**1.09E-06**	**4.42E-02**
C14index	57	ARS-BFGL-NGS-53115	26	11528933	*KIF20B*	61222	3.10E-06	0.126
C14index	25	ARS-BFGL-NGS-63853	26	11942868	*MIR2895*	67662	**2.50E-09**	**1.01E-04**
C14index	61	ARS-BFGL-NGS-12381	26	12200948	*LOC100141242*	24477	4.76E-06	0.193
C14index	17	BTB-01908417	26	12268427	*LOC784522*	15486	**1.71E-10**	**6.94E-06**
C14index	67	BTB-01841682	26	12295284	*LOC784522*	42343	8.27E-06	0.336
C14index	84	ARS-BFGL-NGS-41148	26	12364119	*HTR7*	10881	2.32E-05	0.942
C14index	23	Hapmap52817-rs29011969	26	14155229	*HHEX*	29160	**1.34E-09**	**5.45E-05**
C14index	69	ARS-BFGL-NGS-85864	26	14532797	*CYP26A1*	69001	8.38E-06	0.340
C14index	74	ARS-BFGL-NGS-110475	26	15604631	*PLCE1*	Within	1.04E-05	0.421
C14index	58	BTB-00706838	26	15824141	*TBC1D12*	Within	3.31E-06	0.134
C14index	70	BTB-00927439	26	16315378	*CYP2C19*	20815	9.08E-06	0.369
C14index	18	ARS-BFGL-NGS-29299	26	16614068	*PDLIM1/* ***SORBS1***	Within/73061	**2.06E-10**	**8.36E-06**
C14index	49	Hapmap41595-BTA-60800	26	16791783	***SORBS1***	Within	**1.09E-06**	**4.43E-02**
C14index	28	Hapmap58930-rs29010490	26	16822073	***SORBS1***	Within	**5.90E-09**	**2.40E-04**
C14index	59	ARS-BFGL-NGS-106959	26	17225652	*CC2D2B*	Within	3.80E-06	0.154
C14index	63	ARS-BFGL-NGS-113660	26	17246984	*CC2D2B*	Within	5.20E-06	0.211
C14index	71	ARS-BFGL-NGS-25217	26	17307507	*CCNJ*	13017	9.96E-06	0.404
C14index	66	ARS-BFGL-NGS-114539	26	18808408	*SFRP5*	21032	7.36E-06	0.299
C14index	82	ARS-BFGL-NGS-97471	26	18882047	*CRTAC1*	109	2.27E-05	0.921
C14index	34	ARS-BFGL-NGS-41056	26	18906121	*CRTAC1*	Within	**4.78E-08**	**1.94E-03**
C14index	41	ARS-BFGL-NGS-116902	26	18967997	*CRTAC1*	Within	**4.05E-07**	**1.64E-02**
C14index	43	ARS-BFGL-NGS-25126	26	18994785	*CRTAC1*	Within	**5.96E-07**	**2.42E-02**
C14index	62	ARS-BFGL-NGS-71584	26	20290497	*GOT1/* ***SCD1***	Within/842247	5.11E-06	0.207
C14index	14	ARS-BFGL-NGS-23064	26	20365711	*NKX2-3/* ***SCD1***	34516/767033	**2.95E-11**	**1.20E-06**
C14index	15	ARS-BFGL-NGS-77668	26	20393457	*NKX2-3/* ***SCD1***	6770/744488	**6.02E-11**	**2.44E-06**
C14index	33	ARS-BFGL-NGS-2464	26	20444634	*SLC25A28/* ***SCD1***	21194/688110	**3.96E-08**	**1.61E-03**
C14index	19	BTB-00930925	26	20474308	*SLC25A28/* ***SCD1***	Within/658436	**2.61E-10**	**1.06E-05**
C14index	24	ARS-BFGL-NGS-39397	26	20716721	*DNMBP/* ***SCD1***	Within/416023	**1.54E-09**	**6.25E-05**
C14index	22	BTB-00930720	26	20903573	*LOC511498/* ***SCD1***	Within/244744	**6.89E-10**	**2.80E-05**
C14index	31	Hapmap46411-BTA-15820	26	20984335	***CHUK*** */* ***SCD1***	Within/148409	**1.10E-08**	**4.45E-04**
C14index	16	Hapmap31825-BTA-158647	26	21056547	*PKD2L1/* ***SCD1***	Within/76197	**1.56E-10**	**6.35E-06**
C14index	10	Hapmap33073-BTA-162864	26	21180893	***SCD1***	32576	**3.04E-12**	**1.23E-07**
C14index	1	BTB-00931481	26	21226405	*WNT8B/* ***SCD1***	14100/78088	**6.91E-17**	**2.80E-12**
C14index	13	ARS-BFGL-NGS-110077	26	21322557	*HIF1AN/* ***SCD1***	22399/174240	**2.04E-11**	**8.28E-07**
C14index	11	ARS-BFGL-NGS-108305	26	21363670	***SCD1*** */HIFIAN*	63512/215353	**6.46E-12**	**2.62E-07**
C14index	5	BTB-00931586	26	21409429	*PAX2/* ***SCD1***	61334/261112	**3.39E-13**	**1.38E-08**
C14index	4	ARS-BFGL-NGS-114149	26	21702714	*LZTS2/* ***SCD1***	656/564769	**3.10E-13**	**1.26E-08**
C14index	9	ARS-BFGL-NGS-116481	26	21977581	*LOC100847491/* ***SCD1***	10062/829264	**1.20E-12**	**4.87E-08**
C14index	7	Hapmap24832-BTA-138805	26	22016380	*BTRC/* ***SCD1***	Within/868063	**6.31E-13**	**2.56E-08**
C14index	6	ARS-BFGL-NGS-6259	26	22059103	*BTRC/* ***SCD1***	Within/910786	**5.97E-13**	**2.42E-08**
C14index	8	BTB-00932332	26	22118554	*BTRC/* ***SCD1***	Within/970237	**7.55E-13**	**3.06E-08**
C14index	2	ARS-BFGL-NGS-107403	26	22889812	***NFKB2***	1586	**2.62E-15**	**1.06E-10**
C14index	21	Hapmap48222-BTA-122240	26	23641881	*C26H10orf26*	Within	**5.68E-10**	**2.31E-05**
C14index	30	Hapmap49372-BTA-91009	26	23689229	*C26H10orf26*	3017	**6.47E-09**	**2.63E-04**
C14index	32	BTA-60918-no-rs	26	23853334	*CNNM2*	Within	**3.34E-08**	**1.36E-03**
C14index	12	BTA-60935-no-rs	26	23876476	*CNNM2*	Within	**1.98E-11**	**8.03E-07**
C14index	40	ARS-BFGL-NGS-2180	26	24477962	*SH3PXD2A*	Within	**3.20E-07**	**1.30E-02**
C14index	29	ARS-BFGL-NGS-1092	26	24531763	*SH3PXD2A*	Within	**6.21E-09**	**2.52E-04**
C14index	65	ARS-BFGL-NGS-18194	26	24575207	*SH3PXD2A*	Within	5.58E-06	0.227
C14index	3	ARS-BFGL-NGS-118189	26	24786731	*SLK*	Within	**6.55E-14**	**2.66E-09**
C14index	46	UA-IFASA-4715	26	25314352	*CCDC147*	27285	**9.22E-07**	**3.74E-02**
C14index	44	BTB-00935537	26	26585557	*SORCS3/* ***ECHS1***	187266/719949	**7.48E-07**	**3.04E-02**
C14index	37	Hapmap28763-BTA-162328	26	26757136	*SORCS3/* ***ECHS1***	358845/891528	**7.79E-08**	**3.16E-03**
C14index	60	BTA-87355-no-rs	26	27251857	*SORCS1*	558341	4.71E-06	0.191
C14index	45	ARS-BFGL-NGS-1448	27	37357125	*HOOK3/* ***AGPAT6***	Within/1128138	**7.66E-07**	**3.11E-02**
C18index	11	ARS-BFGL-NGS-110992	28	20421361	*REEP3*	614577	**9.76E-07**	**3.96E-02**
C18index	18	ARS-BFGL-NGS-4865	28	28011033	*CDH23*	Within	2.38E-06	0.097
C18index	86	ARS-BFGL-NGS-12970	29	40646639	*PPP1R32/* ***FADS1***	2220/292226	1.94E-05	0.788
C18index	57	ARS-BFGL-NGS-11681	X	3622615	*SLC25A43*	24203	9.32E-06	0.378
C18index	67	Hapmap48540-BTA-97806	X	8700029	*ODZ1*	Within	1.23E-05	0.498
C18index	70	Hapmap50046-BTA-58882	X	82022276	*MIR374B*	1635	1.33E-05	0.540
C18index	17	Hapmap60551-rs29017241	X	82281306	*XIST*	Within	2.17E-06	0.088
C18index	38	Hapmap60664-rs29017374	X	107043386	*CASK*	113889	4.61E-06	0.187
C18index	66	Hapmap49563-BTA-30596	X	120716539	*IL1RAPL1*	Within	1.21E-05	0.490

Note: see note to Table 2.

**Table 6:** Genome-wise and suggestive significant SNPs for sum of fatty acid traits.

Trait	Rank^b^	SNP	Chr.	Position(bp)	Nearest Gene/Candidate Gene	Distance(bp)	Raw P_value^a^	P_value Bonferroni
SFA	13	Hapmap42233-BTA-49670	1	82559884	*C1H3orf70/* ***EHHADH***	14106/38733	6.58E-06	0.267
SFA/UFA	14	Hapmap42233-BTA-49670	1	82559884	*C1H3orf70/* ***EHHADH***	14106/38733	4.96E-06	0.202
UFA	33	ARS-BFGL-NGS-13938	2	111650859	***MOGAT1*** */* ***ACSL3***	19295/146311	2.25E-05	0.912
SFA	8	Hapmap42304-BTA-73062	5	5049476	*KRR1/* ***OSBPL8***	14567/770655	5.51E-06	0.224
SFA/UFA	12	Hapmap42304-BTA-73062	5	5049476	*KRR1/* ***OSBPL8***	14567/770655	4.17E-06	0.169
UFA	28	Hapmap42304-BTA-73062	5	5049476	*KRR1/* ***OSBPL8***	14567/770655	2.02E-05	0.819
SFA/UFA	32	ARS-BFGL-NGS-38038	5	27992179	*NR4A1*	Within	1.80E-05	0.731
UFA	26	ARS-USMARC-624	5	28859701	*CSRNP2*	Within	1.87E-05	0.760
SFA	10	ARS-BFGL-NGS-8796	5	29095603	*LOC510716*	Within	6.04E-06	0.245
SFA/UFA	8	ARS-BFGL-NGS-8796	5	29095603	*LOC510716*	Within	3.04E-06	0.123
UFA	3	ARS-BFGL-NGS-8796	5	29095603	*LOC510716*	Within	1.44E-06	0.058
SFA/UFA	22	ARS-BFGL-NGS-35179	5	34325053	*SCAF11*	62336	1.21E-05	0.493
UFA	20	ARS-BFGL-NGS-35179	5	34325053	*SCAF11*	62336	1.43E-05	0.579
SFA	17	ARS-BFGL-NGS-69056	5	42285835	*CPNE8*	135184	1.11E-05	0.449
SFA/UFA	29	ARS-BFGL-NGS-69056	5	42285835	*CPNE8*	135184	1.62E-05	0.657
UFA	8	ARS-BFGL-NGS-69056	5	42285835	*CPNE8*	135184	4.25E-06	0.173
UFA	27	Hapmap52463-rs29025831	5	45473334	*LOC101905276*	65002	1.93E-05	0.784
SFA	11	Hapmap39862-BTA-74478	5	85672503	*BCAT1*	74712	6.22E-06	0.253
SFA/UFA	6	Hapmap39862-BTA-74478	5	85672503	*BCAT1*	74712	2.26E-06	0.092
UFA	4	Hapmap39862-BTA-74478	5	85672503	*BCAT1*	74712	1.92E-06	0.078
SFA/UFA	16	ARS-BFGL-NGS-99256	5	104714350	*VWF/* ***OLR1***	Within/4458707	5.47E-06	0.222
UFA	12	ARS-BFGL-NGS-99256	5	104714350	*VWF/* ***OLR1***	Within/4458707	6.47E-06	0.263
UFA	16	BTB-00246150	6	20993424	*PPA2*	Within	9.44E-06	0.383
SFA/UFA	34	Hapmap26001-BTC-038813	6	44926243	***PPARGC1A***	Within	1.97E-05	0.799
UFA	25	Hapmap26001-BTC-038813	6	44926243	***PPARGC1A***	Within	1.85E-05	0.750
UFA	31	BTB-00316291	7	64892251	*SPARC*	Within	2.18E-05	0.887
SFA	15	BTB-00316348	7	64939808	*ATOX1*	5940	8.62E-06	0.350
SFA/UFA	17	BTB-00316348	7	64939808	*ATOX1*	5940	6.58E-06	0.267
UFA	15	BTB-00316348	7	64939808	*ATOX1*	5940	9.25E-06	0.375
SFA	9	BTB-00316650	7	65098028	*GLRA1*	Within	6.02E-06	0.245
SFA/UFA	18	BTB-00316650	7	65098028	*GLRA1*	Within	7.85E-06	0.319
UFA	17	BTB-00316650	7	65098028	*GLRA1*	Within	9.98E-06	0.405
UFA	30	BTB-02040446	8	30320626	*NFIB*	164574	2.16E-05	0.878
UFA	22	Hapmap50126-BTA-83733	9	55449737	*LOC101907134*	18763	1.73E-05	0.702
SFA	24	BTB-01866513	10	70231445	*SLC35F4*	Within	1.73E-05	0.701
SFA	29	BTB-01203179	10	72694329	*DHRS7*	53135	2.03E-05	0.825
SFA/UFA	28	BTB-01203179	10	72694329	*DHRS7*	53135	1.61E-05	0.653
UFA	10	BTB-01203179	10	72694329	*DHRS7*	53135	5.18E-06	0.210
SFA	2	BTB-01332998	10	73466092	*SLC38A6*	15901	**8.08E-08**	**3.28E-03**
SFA/UFA	2	BTB-01332998	10	73466092	*SLC38A6*	15901	**3.54E-07**	**1.44E-02**
UFA	2	BTB-01332998	10	73466092	*SLC38A6*	15901	**4.87E-07**	**1.98E-02**
SFA	1	ARS-BFGL-NGS-4783	10	73487550	*SLC38A6*	37359	**6.07E-08**	**2.46E-03**
SFA/UFA	1	ARS-BFGL-NGS-4783	10	73487550	*SLC38A6*	37359	**2.64E-07**	**1.07E-02**
UFA	1	ARS-BFGL-NGS-4783	10	73487550	*SLC38A6*	37359	**4.01E-07**	**1.63E-02**
SFA	7	BTB-01501723	10	73655817	*TMEM30B*	6161	5.31E-06	0.216
SFA/UFA	37	BTB-01501723	10	73655817	*TMEM30B*	6161	2.28E-05	0.924
UFA	34	BTB-01501723	10	73655817	*TMEM30B*	6161	2.25E-05	0.912
SFA	19	BTB-01079278	11	57078447	*REG3A*	435723	1.39E-05	0.565
SFA/UFA	36	BTB-01079278	11	57078447	*REG3A*	435723	2.12E-05	0.859
SFA	23	BTB-01079350	11	57107070	*REG3A*	464346	1.66E-05	0.675
SFA	3	BTA-119672-no-rs	11	102911946	*AK8*	Within	1.34E-06	0.054
SFA/UFA	10	BTA-119672-no-rs	11	102911946	*AK8*	Within	3.77E-06	0.153
UFA	7	BTA-119672-no-rs	11	102911946	*AK8*	Within	4.03E-06	0.164
UFA	18	BTB-01236909	12	50295112	*TBC1D4*	382864	1.17E-05	0.476
UFA	38	BTB-01980482	12	50451289	*TBC1D4*	226687	2.38E-05	0.964
SFA/UFA	31	ARS-BFGL-NGS-70206	13	48622655	*FERMT1*	Within	1.65E-05	0.670
UFA	37	ARS-BFGL-NGS-70206	13	48622655	*FERMT1*	Within	2.31E-05	0.937
SFA/UFA	25	ARS-BFGL-NGS-57820	14	1651311	*LOC100294916/* ***DGAT1***	Within/144114	1.41E-05	0.572
SFA	5	ARS-BFGL-NGS-4939	14	1801116	***DGAT1***	Within	2.80E-06	0.114
SFA/UFA	3	ARS-BFGL-NGS-4939	14	1801116	***DGAT1***	Within	**1.15E-06**	**4.65E-02**
UFA	9	ARS-BFGL-NGS-4939	14	1801116	***DGAT1***	Within	4.78E-06	0.194
SFA/UFA	21	ARS-BFGL-NGS-107379	14	2054457	*LOC786966/* ***DGAT1***	460/249619	1.17E-05	0.475
SFA/UFA	13	ARS-BFGL-NGS-100480	14	4364952	*TRAPPC9*	Within	4.68E-06	0.190
UFA	14	ARS-BFGL-NGS-100480	14	4364952	*TRAPPC9*	Within	7.52E-06	0.305
SFA	16	ARS-BFGL-NGS-113293	14	77274386	*SLC2A5*	3495	1.03E-05	0.419
SFA/UFA	5	ARS-BFGL-NGS-113293	14	77274386	*SLC2A5*	3495	1.41E-06	0.057
UFA	19	ARS-BFGL-NGS-113293	14	77274386	*SLC2A5*	3495	1.36E-05	0.551
SFA	22	ARS-BFGL-NGS-15481	18	56611355	*AP2A1/* ***SPHK2***	Within/889940	1.55E-05	0.630
SFA/UFA	20	ARS-BFGL-NGS-15481	18	56611355	*AP2A1/* ***SPHK2***	Within/889940	9.71E-06	0.394
UFA	24	ARS-BFGL-NGS-15481	18	56611355	*AP2A1/* ***SPHK2***	Within/889940	1.79E-05	0.725
SFA	20	ARS-BFGL-NGS-75390	19	4286783	*LOC790351*	6533	1.44E-05	0.586
SFA/UFA	19	ARS-BFGL-NGS-75390	19	4286783	*LOC790351*	6533	9.64E-06	0.391
SFA	6	ARS-BFGL-NGS-87368	19	7762820	*C19H17orf67*	67914	3.15E-06	0.128
SFA/UFA	9	ARS-BFGL-NGS-87368	19	7762820	*C19H17orf67*	67914	3.72E-06	0.151
UFA	13	ARS-BFGL-NGS-87368	19	7762820	*C19H17orf67*	67914	6.64E-06	0.270
SFA	21	ARS-BFGL-NGS-118339	20	3347138	*C20H5orf50*	150804	1.54E-05	0.627
SFA/UFA	15	ARS-BFGL-NGS-118339	20	3347138	*C20H5orf50*	150804	5.31E-06	0.215
UFA	23	ARS-BFGL-NGS-118339	20	3347138	*C20H5orf50*	150804	1.75E-05	0.709
SFA/UFA	24	BTA-50482-no-rs	20	36336225	*EGFLAM*	157753	1.35E-05	0.548
UFA	36	BTA-50482-no-rs	20	36336225	*EGFLAM*	157753	2.31E-05	0.936
SFA	14	ARS-BFGL-NGS-116806	20	36450009	*GDNF*	180881	7.68E-06	0.312
SFA/UFA	11	ARS-BFGL-NGS-116806	20	36450009	*GDNF*	180881	3.92E-06	0.159
UFA	11	ARS-BFGL-NGS-116806	20	36450009	*GDNF*	180881	5.23E-06	0.212
SFA	30	ARS-USMARC-Parent-DQ990835-rs29012811	20	36570529	*GDNF*	60361	2.31E-05	0.939
SFA/UFA	27	ARS-USMARC-Parent-DQ990835-rs29012811	20	36570529	*GDNF*	60361	1.56E-05	0.632
UFA	35	ARS-USMARC-Parent-DQ990835-rs29012811	20	36570529	*GDNF*	60361	2.29E-05	0.931
SFA	27	ARS-BFGL-NGS-17676	20	39017985	***PRLR***	55261	1.95E-05	0.790
SFA/UFA	23	ARS-BFGL-NGS-17676	20	39017985	***PRLR***	55261	1.29E-05	0.523
UFA	39	ARS-BFGL-NGS-17676	20	39017985	***PRLR***	55261	2.45E-05	0.994
SFA/UFA	38	ARS-BFGL-NGS-55739	20	39787788	*C1QTNF3*	Within	2.38E-05	0.965
SFA	25	BTB-00783271	20	41201777	*SUB1*	17036	1.73E-05	0.702
SFA/UFA	26	BTB-00783271	20	41201777	*SUB1*	17036	1.54E-05	0.626
UFA	29	BTB-00783271	20	41201777	*SUB1*	17036	2.09E-05	0.850
SFA	12	BTB-01583562	20	55425112	*LOC101905359*	Within	6.28E-06	0.255
SFA	4	Hapmap44836-BTA-51861	21	20990602	*ABHD2/* ***PLIN1***	8117/512222	1.76E-06	0.071
SFA/UFA	4	Hapmap44836-BTA-51861	21	20990602	*ABHD2/* ***PLIN1***	8117/512222	1.39E-06	0.056
UFA	6	Hapmap44836-BTA-51861	21	20990602	*ABHD2/* ***PLIN1***	8117/512222	2.09E-06	0.085
UFA	32	Hapmap35708-SCAFFOLD316799_27843	21	25136856	*SH3GL3*	52052	2.21E-05	0.896
SFA/UFA	39	Hapmap33890-BES3_Contig418_1154	23	40236175	*ATXN1*	Within	2.39E-05	0.968
SFA	28	ARS-BFGL-BAC-28144	25	2606575	*LOC788915*	21466	1.96E-05	0.795
SFA/UFA	7	ARS-BFGL-BAC-28144	25	2606575	*LOC788915*	21466	2.89E-06	0.117
UFA	5	ARS-BFGL-BAC-28144	25	2606575	*LOC788915*	21466	2.06E-06	0.083
SFA	26	BTA-61650-no-rs	26	41850719	*FGFR2*	Within	1.91E-05	0.776
SFA/UFA	35	BTA-61650-no-rs	26	41850719	*FGFR2*	Within	1.98E-05	0.802
SFA/UFA	33	BTB-01926888	27	16398882	*TRIML2/* ***ACSL1***	303782/2110549	1.96E-05	0.797
UFA	21	BTB-01926888	27	16398882	*TRIML2/* ***ACSL1***	303782/2110549	1.58E-05	0.641
SFA	18	ARS-BFGL-NGS-106901	29	44372611	*SCYL1*	Within	1.22E-05	0.495
SFA/UFA	30	ARS-BFGL-NGS-106901	29	44372611	*SCYL1*	Within	1.63E-05	0.663

Note: see note to Table 2.

**Table 7:** Numbers of significant SNPs with genome-wise and suggestive significance for 18 milk fatty acid traits.

Trait	Genome-wise level	Suggestive level	Total
C10:0	1	20	21
C12:0	2	20	22
C14:0	1	26	27
C14:1	34	33	67
C16:0	0	18	18
C16:1	0	6	6
C18:0	13	92	105
C18:1n9c	0	20	20
C18:2n6c	3	3	6
CLA	0	7	7
C14 index	49	35	84
C16 index	0	10	10
C18 index	14	79	93
SFA	2	28	30
UFA	2	37	39
SFA/UFA	3	36	39
C20:0	0	2	2
C22:0	1	0	1
Sum	125	472	597

Note: Associations with C8:0, C18:3n3, C18:3n6 and C20:5n3 only reached chromosome-wise significance or non-significant, so they were not listed.

### Short- and medium-chain saturated fatty acid traits (SCFA and MCFA)

For C10:0, C12:0 and C14:0, 21, 22 and 27 SNPs were detected, respectively. Of these 70 SNPs, 10 were associated with two or three traits. The most significant association of C10:0 (*P* = 5.89E-07), C12:0 (*P* = 3.94E-07), and C14:0 (*P* = 1.58E-07) were identified with BTB-01556197 on BTA9, BTA-76414-no-rs on BTA21 and Hapmap49848-BTA-106779 on BTA5, respectively. The SNP strongly associated with C10:0 (*P* = 8.54E-06), C12:0 (*P* = 1.16E-07) and C14:0 (*P* = 6.01E-06), ARS-BFGL-NGS-39328, is 58,172 bp close to the *fatty acid synthase* (*FASN*) gene on BTA19, which is well-known to affect fat composition of dairy cattle and beef.

### Long-chain saturated fatty acid traits (LCFA)

A total of 126 significant SNPs for LCFA were detected mainly on BTA1, 2, 8, 10 and 17, including 105 for C18:0, 18 for C16:0, 2 for C20:0 and one for C22:0. The top one significant SNP (ARS-BFGL-BAC-13788) was associated with C18:0 (*P* = 9.17E-09) on BTA12. The strongest association of C22:0 (*P* = 6.70E-07) was identified with the SNP (ARS-BFGL-NGS-109692) on BTA1. The SNP (ARS-BFGL-NGS-4939) associated with C16:0 (*P* = 9.04E-06) on BTA14 is located within the *diacylglycerol O-acyltransferase 1*(*DGAT1*) gene, the major gene with large effect on milk fat in dairy cattle.

### Monounsaturated and polyunsaturated fatty acid traits (MUFA and PUFA)

A total of 93 and 13 Significant SNPs for MUFA and PUFA were detected, respectively. Of them, 67, 6 and 20 SNPs were associated with C14:1, C16:1 and C18:1n9c, respectively. For C14:1, 29 out of 34 genome-wise significant SNPs on BTA26 were clustered within three regions: 6 fell in a 6.40 Mbp region (10.39∼16.79 Mbp), 16 fell in a 1.75 Mbp region (20.36∼22.11 Mbp) containing the *stearoyl-CoA desaturase* (*SCD1*) gene, and 7 SNPs fell in a 4.37 Mbp region (22.88∼27.25 Mbp). The SNP (BTB-00931481) on BTA26, 78,088 bp near to the *SCD1* gene, showed the strongest effect (*P* = 7.08E-13). Though no SNPs for C16:1 and C18:1n9c reached genome-wise level, the second suggestive significant SNP (ARS-BFGL-NGS-4939) for C18:1n9c (*P* = 3.01E-06) is located within the *DGAT1* gene on BTA14.

As for PUFA, 6 and 7 significant SNPs were detected for C18:2n6c and CLA, respectively. The most significant SNP (ARS-BFGL-NGS-4939) associated with C18:2n6c (*P* = 6.87E-08) is located within the *DGAT1* gene on BTA14, while the most significant SNP (BTA-50525-no-rs) for CLA only reached suggestive level (*P* = 1.16E-05).

### Indices of fatty acid traits (C14 index, C16 index, C18 index)

For indices of C14, C16 and C18, totally 84, 10 and 93 significant SNPs were detected, respectively. Forty-two SNPs associated with C14 index are located within a region of 16.89 Mbp on BTA26, which included four small segments: 6 in a 2.40 Mbp segment (9.86∼12.26 Mbp), 7 in a 4.84 Mbp segment (14.15∼18.99 Mbp), 18 in an 1.75 Mbp segment (20.36∼22.11 Mbp) containing the *SCD1* gene, and 11 in a 3.87 Mbp segment (22.88∼26.75 Mbp). In addition, 56 common SNPs for C14 index and C14:1, 4 common SNPs for C18 index and C18:1n9c, 28 common SNPs for C18 index and C18:0, and 4 common SNPs for C16 index and C16:1were identified.

### Sum of fatty acid traits (SFA, UFA, SFA/UFA)

A total of 108 significant associations mainly on BTA5, 10 and 20 with three sum of fatty acid traits were detected, which involved 52 distinct SNPs. Of them, 22 SNPs were simultaneously associated with three traits and 12 were common for two traits. The 0.96 Mbp region (72.69–73.65 Mbp) on BTA10 was associated with the three traits, in which the SNP (ARS-BFGL-NGS-4783) showed the strongest association for SFA (*P* = 6.07E-08), UFA (*P* = 4.01E-07) and SFA/UFA (*P* = 2.64E-07), respectively.

## Discussions

To our knowledge, this is one of the first GWA study for milk fatty acids with high density SNP Chip. In this study, we detected a total of 83 genome-wise and 314 suggestive significant SNPs for 22 fatty acid traits. Among them, some SNPs are located within the QTL regions on BTA6, 14, 19 and 26 those have been reported by Stoop *et al* [13], Schennink*et al* [14] and Morris *et al* [30] for bovine milk fat composition. Sixteen SNPs on BTA14, 5 SNPs on BTA19, and 5 SNPs on BTA7 were consistent with the previous GWA study for fatty acid traits of dairy cattle [24]. However, associations of BTA19 with C16:1 and CLA were not found in this study. This is probably due to a different dairy population was tested. Several SNPs were found to be located within and/or close to genes that are known to have functions related to the milk composition. In addition, 20 novel prospective candidate genes affecting milk fatty acid traits were identified.

### Chromosomes underlying novel promising candidate genes

On BTA1, 23 SNPs associated with 9 fatty acids (C10:0, C12:0, C14:0, CLA, C18:0, C18 index, C22:0, SFA and SFA/UFA) were detected. The SNP associated with SFA and SFA/UFA is 38,733 bp away from the *3-hydroxyacyl Coenzyme A dehydrogenase* (*EHHADH*) gene. As a bi-functional enzyme, EHHADH is part of the classical peroxisomal fatty acid β-oxidation pathway, which is highly inducible via peroxisome proliferator-activated receptor α (PPARα) activation [31] and is essential for the production of medium-chain dicarboxylic acids [32]. Four SNPs for C18:0 and C18 index form an 0.40 Mbp region containing the *1-acylglycerol -3- phosphate O-acyltransferase 3* (*AGPAT3*) gene. AGPAT catalyzes the first step during de novo synthesis of triacylglycerol. AGPAT3 is a member of the acyltransferase family [33] and plays a key role in de novo phospholipid biosynthetic due to its function of converting lysophosphatidic acid into phosphatidic acid [34].

On BTA2, 21 SNPs showed associations with 7 traits (C10:0, C12:0, C14:0, C14:1, C18:0, C18 index and UFA). Two SNPs associated with C18:0 and C18 index are 0.50 Mbp away from the *signal transducer and activator of transcription 1* (*STAT1*) gene, especially, one of them is the top 3 significant SNP for C18 index. STATs are transcription factors known to importance to cytokine signaling. STAT1 has a role in regulating the transcription of genes involved in milk protein synthesis and fat metabolism in Holstein [35]. The SNP associated with UFA is 0.14 Mbp and 19,295 bp away from the *acyl-CoA synthetase long-chain family member 3* (*ACSL3*) gene and the *monoacylglycerol O-acyltransferase 1* (*MOGAT1*) gene, respectively. ACSL3 is an isozyme of the long chain fatty acids coenzyme A ligase family that convert free long chain fatty acids into fatty acyl-CoA esters and has a substrate preference for PUFA [36]. Depletion of *ACSL3* by RNAi causes a significant reduction in fatty acids uptake, thereby plays a key role in lipid biosynthesis and fatty acids degradation [37]. MOGAT1 catalyzes the synthesis of diacylglycerols, the precursor of triacylglycerol and phospholipids [38]. Two SNPs associated with C18:0 and C18 index are 5.70 Mbp and 5.74 Mbp away from the *fatty acid binding protein 3* (*FABP3*) gene, respectively. FABP3 provides fatty acids for SCD, which is one of specific transporters for LCFA and one of the most abundant isoforms in bovine mammary tissue [39]. Eight contiguous SNPs associated with C18:0 and C18 index are located within a chromosome region of 63.58∼98.16 Mbp that overlaps a reported QTL region (67.56∼68.25 Mbp) for C14 index, C16 index, C18 index, SFA, MUFA, PUFA and SFA/UFA [40].

On BTA5, 17 SNPs showed association with 9 traits (C10:0, C14:0, C16:0, C18:0, C18:1n9c, C18 index, SFA, SFA/UFA and UFA). The top one significant SNP for C14:0 is within the *carboxypeptidase M* (*CPM*) gene. Up-regulation of *CPM* in macrophages (MAs) is associated with increased lipid uptake [41] and the highest expression of *CPM* was detected in human adipocyte cell [42]. The SNP associated with C18:1n9c, SFA, UFA and SFA/UFA is 0.77 Mbp away from the *oxysterol binding protein-like 8* (*OSBPL8*) gene which encodes a member of the oxysterol-binding protein (OSBP) family, a group of intracellular lipid receptors. *OSBPL8* has the capacity to modulate lipid homeostasis and *SREBP* activity probably through an indirect mechanism [43] and is a negative regulator of sequestering of triglyceride [44]. The chromosome region of 8.43 Mbp (95.74∼104.17 Mbp) associated with C18:1n9c, C18 index, UFA and SFA/UFA contains the *oxidized low density lipoprotein (lectin-like) receptor 1* (*OLR1*) gene which can bind and degrade oxidized low-density lipoprotein [45], [46].

On BTA9, 13 SNPs showed association with 9 traits (C10:0, C12:0, C14:0, C16:1, C18:0, C18 index, C18:1n9c, CLA and UFA). The top one significant SNP for C10:0 and C12:0 and the SNP for C18:0 are 0.40, 0.81 Mbp away from the *5-hydroxytryptamine (serotonin) receptor 1B* (*HTR1B*) gene, respectively. HTR1B is one of receptors for 5-hydroxytryptamine (serotonin). *HTR1B* gene knocked-out mice showed elevated aggression, higher food intake and impulsivity, indicating it possibly acts as a bridge between behavior and energy homeostasis [47]. Fatty acids, as energy signal, affect the activity of hypothalamic fat-sensitive neurons and impair nervous control of energy homeostasis [48]. *HTR1B* was also shown to affect milk production performance in Chinese Holstein [49].

On BTA20, 30 SNPs showed association with 11 traits (C12:0, C14:0, C14:1, C18:0, C18 index, C18:1n9c, C18:2n6c, CLA, SFA, SFA/UFA and UFA). The SNP associated with C18:0 is located within the *growth hormone receptor* (*GHR*) gene, the well-known major gene affecting milk fat trait [50]. Three SNPs associated with C18:0 and C18 index are within an 1.46 Mbp region containing the *3-oxoacid CoA transferase 1* (*OXCT1*) gene which has a major function to utilize ketone bodies by mammary [39]. The SNP associated with SFA, UFA and SFA/UFA is located within the *prolactin receptor* (*PRLR*) gene which activates the *STAT5A* (*Signal transducer and activator of transcription 5A*) expression [51] and is associated with milk composition traits [52].

On BTA21, 10 SNPs showed association with 8 traits (C10:0, C12:0, C16:0, C18 index, CLA, SFA, SFA/UFA and UFA). The 0.15 Mbp region (9.37∼9.52 Mbp) associated with C10:0 and C12:0 is 1.10 Mbp away from the *insulin-like growth factor 1 receptor* (*IGF1R*) gene. Furthermore, the top one significant SNP for C12:0 is located within such region. *IGF1R* was found to affect milk composition traits [53]. Two SNPs associated with C10:0, SFA, SFA/UFA and UFA are close to the *lipin 1* (*PLIN1*) gene which plays a vital role in regulation on the expression of genes involved in milk fat synthesis [39].

On BTA26, 71 SNPs showed association with 8 traits (C10:0, C12:0, C14:1, C14 index, C16:0, C18:0, SFA and SFA/UFA). The nearest SNP is 32,576 bp close to the *SCD1* gene which encodes key enzyme responsible for the conversion of SFA to MUFA in mammalian adipocytes [54] and were shown to be associated with milk fatty acids [10], [11], [12], [40], [55]. The SNP associated with C10:0 and C14 index is located within the *protein kinase, cGMP-dependent, type I* (*PRKG1*) gene which is a key regulator of adipokine secretion and browning of white fat depots [56] and brown fat cell differentiation [57]. The SNP associated with C12:0 is located within the *multiple inositol-polyphosphate phosphatase 1* (*MINPP1*) gene. *MINPP1* encodes multiple inositol polyphosphate phosphatase which converts 2, 3 bisphosphoglycerate (2,3-BPG) to 2-phosphoglycerate [58]. As known, 2,3-BPG is a key substrate for the triglyceride (TG) synthesis. Two SNPs associated with C14:1 and C14 index are 7,406 bp and 35,288 bp away from the *lipase, family member J* (*LIPJ*) gene and the *lipase, family member K* (*LIPK*) gene, respectively. The two genes belong to lipase family and take part in lipid catabolic process in human [59] and have an essential function in lipid metabolism of the most differentiated epidermal layers. The SNP for C14:1 and the one for C14:1 and C14 index are 0.71 Mbp and 0.89 Mbp away from the *enoyl coenzyme A hydratase, short chain, 1*(*ECHS1*) gene, respectively. ECHS1 takes part in fatty acid biosynthesis, elongation and metabolism and catalyzes the β-oxidation of fatty acid in human [60]. Two SNPs associated with C14:1 and C14 index are located within the *sorbin and SH3 domain containing 1 (SORBS1)* gene. SORBS1 is an important protein in the insulin-signaling pathway in the adipose depots of human [61] and has a positive regulation of lipid biosynthetic process [62]. The SNP associated with C14:1 and C14 index is located within the *conserved helix-loop-helix ubiquitous kinase* (*CHUK*) gene. CHUK takes part in mammary gland alveolus development, mammary gland epithelial cell proliferation [63] and lipogenesis through NF-κB activation pathway [64]. The SNP associated with C14:1 and C14 index is 1,586 bp away from the *nuclear factor of kappa light polypeptide gene enhancer in B-cells 2* (*NFKB2*) gene which is essential for normal development of the mammary gland [65].

### Chromosomes underlying known candidate genes

Apart from the seven chromosomes as mentioned above, other chromosome regions harboring several significant SNPs within or near to known genes involved in fatty acids synthesis were identified.

On BTA14, the SNP associated with 9 traits (C14:1, C16:0, C18:1n9c, C18:2n6c, C14 index, C18 index, SFA, UFA and SFA/UFA) is located within the *DGAT1* gene, which has been confirmed to be the true QTL for milk fat composition in dairy cattle [66]. On BTA19, three SNPs associated with C10:0, C12:0 and C14:0 are 0.05, 1.71 and 1.42 Mbp away from *FASN*, *ACACA (Acetyl-CoA carboxylase alpha)* and *SREBF1 (Sterol regulatory element binding transcription factor 1)*, respectively. FASN is a multifunctional enzyme with a central role in the *de novo* lipogenesis in mammals [45], [67]. ACACA catalyses biosynthesis of LCFA in mammalian cytosol [68]. SREBF1 is a transcription factor that regulates the expression of the *SCD1* gene which is related to several genes of lipid metabolism [69]. On BTA6, the SNP associated with C18index is located within the *peroxisome proliferator-activated receptor gamma, coactivator 1 alpha (PPARGC1A)* gene which is involved in the regulation of fatty acids transcription and mammary gland metabolism [70]. Six SNPs on BTA13 associated with C16:1, C16 index, C18:0 and C18 index are located 1.01∼3.11 Mbp away from the *acyl-CoA synthetase short-chain family member 2* (*ACSS2*) gene. ACSS2 provides activated acetate for *de novo* fatty acids synthesis [39]. Three SNPs within an 2.34 Mbp segment (54.27∼56.61 Mbp) on BTA18 associated with C10:0, SFA, SFA/UFA and UFA harbors the *Sphingosine kinase 2* (*SPHK2*) gene. As a lipid mediator with both intra- and extracellular functions, SPHK2 has diacylglycerol kinase activity and involves the sphingolipid synthesis [39]. Two SNPs on BTA23 associated with C16:0 is located 1.21 Mbp and 0.79 Mbp away from the *butyrophilin, subfamily 1, member A1* (*BTN1A1*) gene, which is essential for milk lipid droplet formation [39], and the *PRL* gene, which impacts milk fat composition through *STAT5A* [45], respectively.

The 0.03 Mbp region (16.39∼16.42 Mbp) on BTA27 associated with C12:0, SFA/UFA and UFA is 2.11 Mbp away from the *acyl-CoA synthetase long-chain family member 1*(*ACSL1*) gene. ACSL1 has a vital role in fatty acids activation for milk TAG [39]. The SNP for C14 index is 1.12 Mbp away from the *1-acylglycerol-3-phosphate O-acyltransferase 6* (*AGPAT6*) gene, a novel lipid biosynthetic gene required for triacylglycerol production in mammary epithelium, if *AGPAT6* was knocked out, lactating mice failed to synthesize milk fat [71]. The SNP on BTA29 associated with C18 index is located 0.29 Mbp away from the *fatty acid desaturase 1*(*FADS1*) gene which catalyzes the synthesis of LCFA [72].

No significant SNPs were detected with C8:0, C18:3n3, C18:3n6 and C20:5n3, probably because these four traits have special population requirements.

## Conclusions

The present genome-wide association study identified 83 genome-wide and 314 suggestive significant SNPs associated with 18 milk fatty acid traits. Some of these SNPs were located within or near to previously reported genes and QTL regions, while some of the SNPs were novel. Consequently, 20 novel promising candidate genes were identified for C10:0, C12:0, C14:0, C14:1, C14 index, C18:0, C18:1n9c, C18 index, SFA, UFA and SFA/UFA, such as *HTR1B, CPM, PRKG1, MINPP1, LIPJ, LIPK, EHHADH, MOGAT1, ECHS1, STAT1, SORBS1, NFKB2, AGPAT3, CHUK, OSBPL8, PRLR, IGF1R, ACSL3, GHR and OXCT1*. Our findings are helpful for follow-up studies to fine-mapping to unravel causal mutations for milk fatty acid traits in dairy cattle.

## Supporting Information

Figure S1 **Manhattan plots for each studied milk fatty acids trait.** BTAX is represented by BTA30), the first line represents genome-wise significant level (raw *P*<1.23E-06), and the second line represents suggestive significant level (raw *P*<2.46E-05).
(PPTX)
